# Designing the cavity architecture in double gate junctionless field effect transistors for enhanced biomolecule detection†

**DOI:** 10.1039/d4na00928b

**Published:** 2025-04-25

**Authors:** Shahriar Khan, Ehsanur Rahman

**Affiliations:** a Department of Electrical and Electronic Engineering, Bangladesh University of Engineering and Technology Dhaka 1000 Bangladesh ehsaneee@eee.buet.ac.bd

## Abstract

This study has investigated double-gate junctionless field effect transistor (DG-JLFET) designs with different cavity configurations and assessed their impact on biosensing performance. Through simulations and analysis of the electrical properties, this study has identified structures that significantly enhance biosensing performance compared to traditional DG-JLFETs. Different cavity architectures have been simulated and evaluated using key biosensing metrics, including the threshold voltage, change in threshold voltage, percentage change in threshold voltage, change in the minimum point of surface potential, *I*_on–off_ ratio, and sensitivity. Analysis of all the structures has revealed that no single structure has outperformed others across all the metrics when the dielectric constant is varied over a wide range. Notably, structure D, featuring drain side cavities, has shown the highest *I*_on–off_ ratio, with values of 3.03 × 10^7^–3.73 × 10^7^ for keratin. In contrast, structure E, with an asymmetrical cavity arrangement featuring an upper cavity on the left and a lower cavity on the right, has exhibited the highest sensitivity, achieving 98.63%–99.25% for the same biomolecule. When considering sensitivity as the key biosensing metric, structures E, F (alternating cavities on the vertical axis), and G (a central upper cavity and bilateral lower cavities) have shown better performance than all the other configurations. This study has further investigated the effect of varying the dielectric constant and channel occupancy of biomolecules on biosensing performance. For the above parametric variations, structure E has shown the highest change in the threshold voltage, while structure G has achieved the highest percentage change in the threshold voltage. These results contribute to the systematic design of DG-JLFET-based biosensors, providing a framework for optimizing cavity architectures to enhance biomolecule detection sensitivity.

## Abstract 2

This study has investigated double gate junctionless field effect transistor (DG-JLFET) designs with different cavity configurations and assessed their impact on biosensing performance.

## Graphical abstract

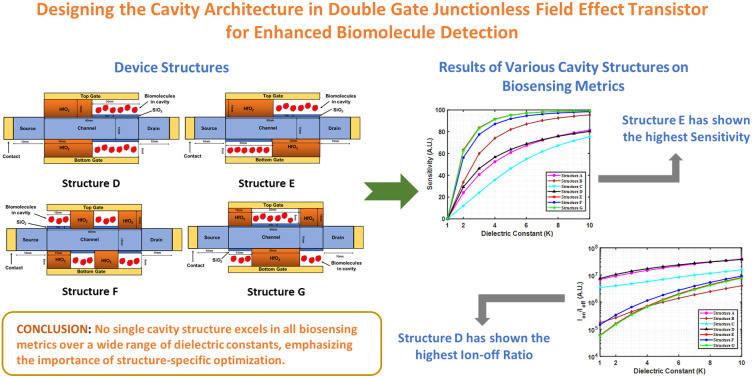

## 1. Introduction

Biosensors are analytical devices that integrate biological components with physicochemical transducers for precise and efficient compound detection across diverse fields. They play a crucial role in the food industry by detecting toxins, harmful chemicals, and viruses to ensure safety and quality control^1^ and are indispensable in biomedical research for studying biomolecular interactions and drug discovery.^2,3^ In forensic science, biosensors facilitate the analysis of DNA, blood, and other biological evidence,^4^ while in medical diagnostics, they enable early disease detection through biomarker identification, as demonstrated during the COVID-19 pandemic.^5^ Additionally, they are vital for environmental monitoring, aiding in the detection of pollutants, water quality assessment, and ecological impact studies, thereby contributing to public health protection.^6^ Biosensors are classified based on their transduction mechanisms, including resonant, optical, thermal, and electrochemical types,^7–11^ with electronic biosensors emerging as key tools for real-time, label-free biomolecular detection. Leveraging low-dimensional materials, these sensors exhibit high sensitivity and specificity, enabling applications in protein dynamics monitoring, DNA hybridization, and antibody–antigen interactions.^12,13^ Advancing electronic biosensor technologies is essential for enhancing diagnostic capabilities, biomedical research, and environmental surveillance, ensuring greater accuracy, efficiency, and real-world applicability.

As discussed, biosensors play a vital role across various fields, with electronic biosensors offering real-time, label-free detection. Field-Effect Transistor (FET)-based biosensors have gained prominence due to their high sensitivity, direct transduction, and CMOS compatibility.^14,15^ The first FET-based biosensor, the Ion-Sensitive Field-Effect Transistor (ISFET), introduced by Bergveld in 1970, enabled charged biomolecule detection but faced challenges in CMOS integration and detecting charge-neutral biomolecules.^16–20^ Dielectric Modulated FETs (DM-FETs) were developed to address these limitations, incorporating vertical nanogaps for improved biomolecule sensing, although they posed structural stability concerns.^21–24^ Recent advancements include junctionless FETs (JLFETs), which exhibit superior short-channel immunity and high *I*_on–off_ ratios, making them ideal for biosensing applications.^25–27^ The emergence of n-type split-gate JLFETs for label-free biomolecule detection under dry conditions further underscores the technological evolution of FET-based biosensors, catering to the growing demand for high-precision diagnostics in medical and environmental monitoring.^28,29^

Existing research has investigated various biosensing structures and proposed alternative design configurations for biomolecule detection. In ref. 30, an asymmetrical channel junctionless FET was introduced, utilizing wide-source narrow-drain and narrow-source wide-drain configurations to enhance sensitivity. A dielectric-modulated junctionless gate stack MOSFET, analyzed in ref. 31, improved detection by incorporating a dielectric stack, while a symmetric split-gate junctionless FET, demonstrated in ref. 32, optimized sensing performance by ensuring uniform electric field distribution. Similarly, in ref. 33, the author explored the integration of nanocavities in a triple-hybrid metal gate-all-around junctionless nanowire FET, enhancing biomolecule immobilization. Further advancements have leveraged novel materials and quantum effects to enhance biosensing capabilities. A quantum ballistic analysis in ref. 34 demonstrated the potential of TMDC-based double-gate junctionless FETs for biomolecule detection in the short-channel regime. In ref. 35, monolayer MoS_2_ and WSe_2_ FETs exhibited superior pH sensing and protein detection sensitivity. Additionally, a gate-all-around junctionless FET, specifically designed for label-free SARS-CoV-2 detection, was introduced in ref. 36.

Recent studies have also focused on biosensing sensitivity and stability. A MoSe_2_ transistor-based biosensor, developed in ref. 37, utilized a pyrene-based supporter molecule to mitigate Debye screening, enhancing streptavidin detection. In ref. 38, a van der Waals bipolar junction transistor biosensor demonstrated high current gain and rapid biomolecule sensing, while ref. 39 investigated a Graphene/WSe_2_ heterostructure, showcasing spin current modulation for potential spintronic biosensing applications. A junctionless nanotube FET for biomolecule detection was explored in ref. 40, while a misaligned double-gate junctionless MOSFET in ref. 41 featured an alternately positioned cavity for improved sensing efficiency. In ref. 42, work function optimization in a dual-material double-gate junctionless MOSFET was employed to enhance sensitivity. Comparative analyses and theoretical modeling continue to refine biosensor performance. A comparative study of DG-MOSFETs with and without gate stacks in ref. 43 provided insights into sensing efficiency, while ref. 44 demonstrated that a trilayer TMDC heterostructure improved drain current characteristics for biomolecule and pH sensing. In ref. 45, an analytical model assessed the influence of biomolecule position and fill factor on dielectric-modulated double-gate junctionless field-effect transistors (DM-DG JLFETs). Finally, ref. 46 developed a compact *I*–*V* model for TMDC-based MOSFET and DM-FET biosensors, improving device performance through transport modeling.

The past literature on DM-FET biosensors has predominantly focused on optimizing specific device structures to enhance sensitivity for improved biomolecule detection. Each study has presented unique device configurations aimed at achieving more accurate and efficient detection by refining and advancing their designs. Various approaches such as different cavity locations, materials, gate lengths, and doping concentrations have been explored to improve the detection process. However, there is a significant research gap regarding the influence of cavity location on the performance of biosensing devices. The current literature lacks comprehensive studies investigating how different cavity locations affect sensitivity, and which cavity architecture is optimal for biomolecule detection. To address this gap, the fundamental purpose of this research is to define multiple cavity structures within a double-gate junctionless field effect transistor (DG-JLFET) and evaluate their performance in detecting biomolecules. By maintaining nearly identical device parameters across all configurations, this investigation will isolate the impact of cavity location on biomolecule detection. This controlled approach will provide clear insights into which cavity location offers the best biosensing performance. The results of this study are expected to contribute significantly to the understanding and design of DG-JLFET-based biosensors, thereby advancing the field of biomolecule detection.

## 2. Methodology and model verification

### 2.1. Working principle of biosensor devices

In these devices, cavities are engineered within the gate oxide to serve as sensing sites for target biomolecule anchoring. Initially air-filled, these cavities exhibit a distinct dielectric constant within the oxide. Upon introducing and immobilizing target biomolecules such as streptavidin, proteins, biotin, enzymes, or APTES, a shift in the dielectric constant occurs, modulating the gate capacitance. Consequently, this alteration influences the electrical properties of the devices, including the drain current, threshold voltage, change in threshold voltage, and the on–off ratio of the drain current (*I*_on–off_).

### 2.2. Device architectures of DM-DG JLFETs studied in this work

**Fig. 1:**
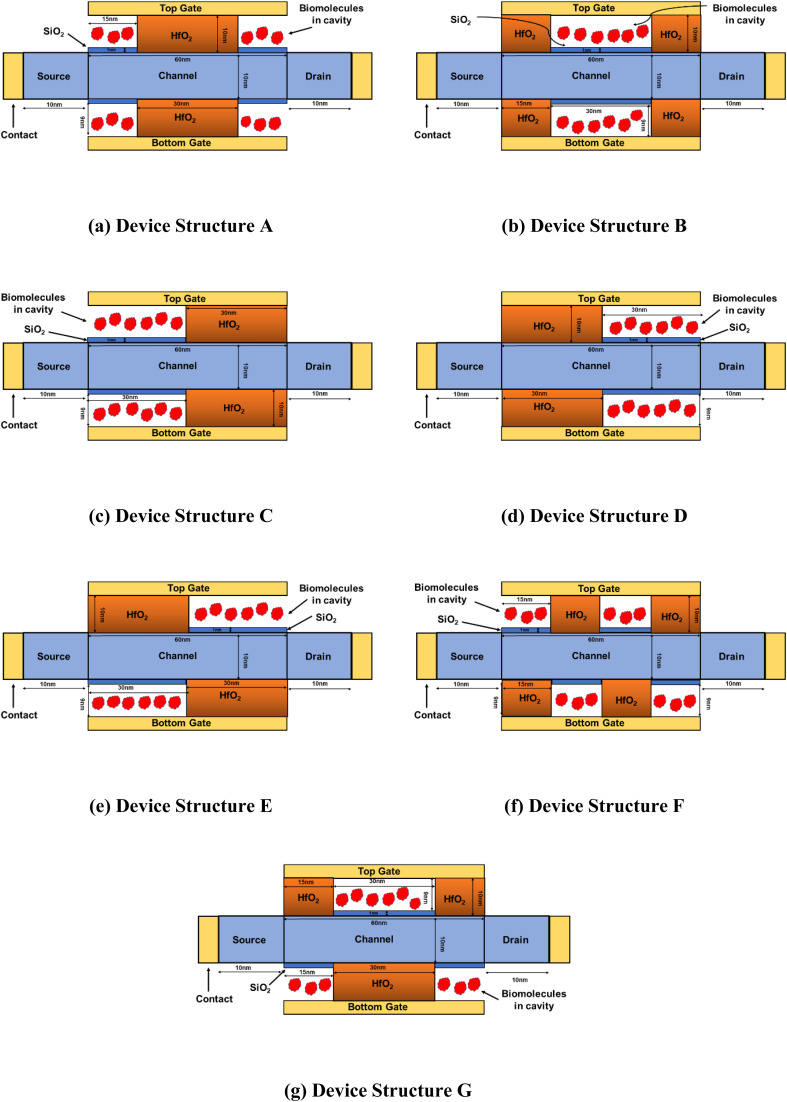
Schematic of biomolecule-filled cavity structures of DM-DG JLFET-based biosensors: (a) Symmetrical cavity structure with a cavity length of 15 nm; (b) middle-positioned cavity structure with a cavity length of 30 nm; (c) left-positioned cavity structure with a cavity length of 30 nm; (d) right-positioned cavity structure with a cavity length of 30 nm; (e) asymmetrical cavity structure with a 30 nm cavity at the upper-right and lower-left positions; (f) alternating upper-lower cavity structure with a cavity length of 15 nm; and (g) alternating upper-lower cavity structure with a 30 nm cavity in the upper part and two 15 nm cavities located at the edges of the lower part. The devices have identical parameters: a cavity thickness of 9 nm, HfO_2_ thickness of 10 nm, SiO_2_ thickness of 1 nm, channel thickness of 10 nm, doping concentration of 3 × 10^18^ cm^−3^, channel length of 60 nm, and drain & source length of 10 nm.

Fig. 1(a)–(g) show the different n-type DM-DG JLFET-based biosensor structures studied in this work, each featuring a gate length of 60 nm and a total cavity length of 30 nm. The high-permittivity dielectric HfO_2_ separates the cavities under the gate, with each cavity differing by the dielectric constant (*K*). The source and drain lengths are set at 10 nm to reduce parasitic resistance effects.^47^ The study considers charge-neutral biomolecules such as streptavidin (*K* = 2.1), protein (*K* = 2.5), biotin (*K* = 2.63), APTES (*K* = 3.57), gluten (*K* = 7), and keratin (*K* = 8–10).^45,48,49^

Considering the S-protein and C-DNA present in SARS-COV-2, the values of the dielectric constant used to represent them are 4 and 10.^50^

Table 1 lists the thickness and dielectric constant values of biomolecules. The channel portion in the cavity region is coated with a native 1 nm SiO_2_ oxide layer to ensure reliable isolation between the active region and the biomolecules and to ensure realistic device modeling, influencing the electrical properties and biomolecule interactions.^54^ The cavity and gate oxide thicknesses are 9 and 10 nm, respectively. The Si-film, drain, and source regions maintain a uniform doping concentration of *N*_D_ = 3 × 10^18^ cm^−3^.

**Table 1:** Biomolecular thickness and dielectric constant values^51–53^

Biomolecules name	Bio thickness	Dielectric constant (*K*)
Streptavidin	6.1 nm	2.1
Biotin	0.6 nm	2.63
APTES	0.9 nm	3.57
Protein		2.5
Gluten		7
Zein		5–7
Keratin		8–10
SARS-COV-2		4, 10

Tables 2 and 3 present the device and material parameter values, respectively, used in this study.

**Table 2:** The values of device parameters

Device parameters	Values (nm)
Gate length	60
Total cavity length	30
Source and drain length	10
Thickness of SiO_2_	1
Thickness of HfO_2_	10
Thickness of cavity	9
Channel length	60
Thickness of channel	10

**Table 3:** The values of material parameters

Material parameters	Values
Gate work function	4.88 eV
Doping concentration	3 × 10^18^ cm^−3^

### 2.3. Simulation methodology

The simulation study was conducted using Silvaco TCAD, a widely used Technology Computer-Aided Design software to analyze the electrical characteristics and performance of the DM-DG JLFET structures. This tool solves Poisson's equation and drift-diffusion transport equations through finite-element numerical methods, enabling accurate modeling of the electrical characteristics and performance of DM-DG JLFET structures. The process begins by defining the mesh and structure based on the device parameters. Then, the appropriate model and simulation method are selected to simulate the device structure. Previous studies have demonstrated consistent results using this tool, further supporting its reliability.^55–57^ In this study, the simulations have employed the Lombardi model, concentration-dependent mobility, Boltzmann transport statistics, and bandgap narrowing to investigate drain current and device behavior. Also, the concentration-, voltage-, and temperature-dependent model has been used to assess carrier mobility and its dependence on electric fields and doping concentration.^58^ The Shockley–Read–Hall model has been employed to account for additional energy balance, which can be established through energy exchange between carriers and the lattice. Quantum effects and ballistic transport have not been considered in the simulation since the channel thickness is greater than 10 nm.^59^

### 2.4. Calculation of biosensing metrics

In the context of cavity architecture design in DG-JLFETs for improved biomolecule detection, several key biosensing metrics have been defined to evaluate and compare the electrical responses of different structures. These metrics include the threshold voltage, change in threshold voltage, percentage change in threshold voltage, *I*_on–off_ ratio, and sensitivity. Through an analysis of these parameters, the performance and efficacy of DG-JLFETs in biomolecule detection can be evaluated, thereby ensuring that the designed architecture fulfills the prerequisites for precise and dependable biosensing applications.

The sensitivity parameter is used to quantify the responsiveness of a system or device to changes in specific inputs or stimuli. High sensitivity indicates that the device is highly responsive to changes in the input signal. The sensitivity parameter is an important figure of merit for many devices, as it determines the minimum detectable signal and the device's overall performance. For the specific device structures investigated, sensitivity has been evaluated in the following manner.1
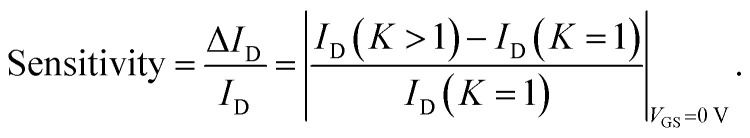


The threshold voltage (*V*_th_) has been determined using the constant-current method.^60^ The gate voltage at which the drain current reaches 10^−7^ A μm^−1^ has been extracted and subsequently defined as the threshold voltage.^61^ The absolute change in threshold voltage and the percentage change in threshold voltage have been determined using the following approaches.

2 Change in threshold voltage = |*V*_th_(*K* > 1) − *V*_th_(*K* = 1)|.

3
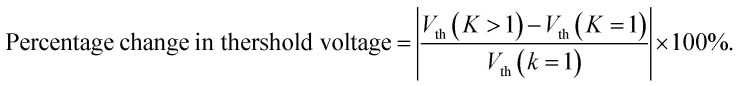


The drain current has been calculated in both the off and on states to determine the *I*_on–off_ ratio. The on-state current (*I*_on_) has been calculated at *V*_GS_ = 1 V, whereas the off-state current (*I*_off_) has been calculated at *V*_GS_ = 0 V, with a drain voltage *V*_DS_ of 1 V in both cases. The following formula has been used in this evaluation.4
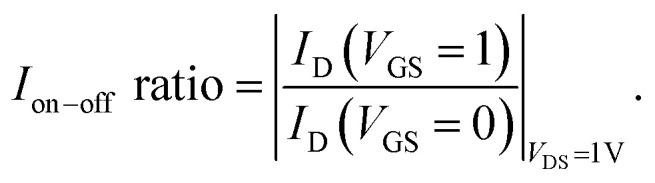


### 2.5. Benchmarking

**Fig. 2:**
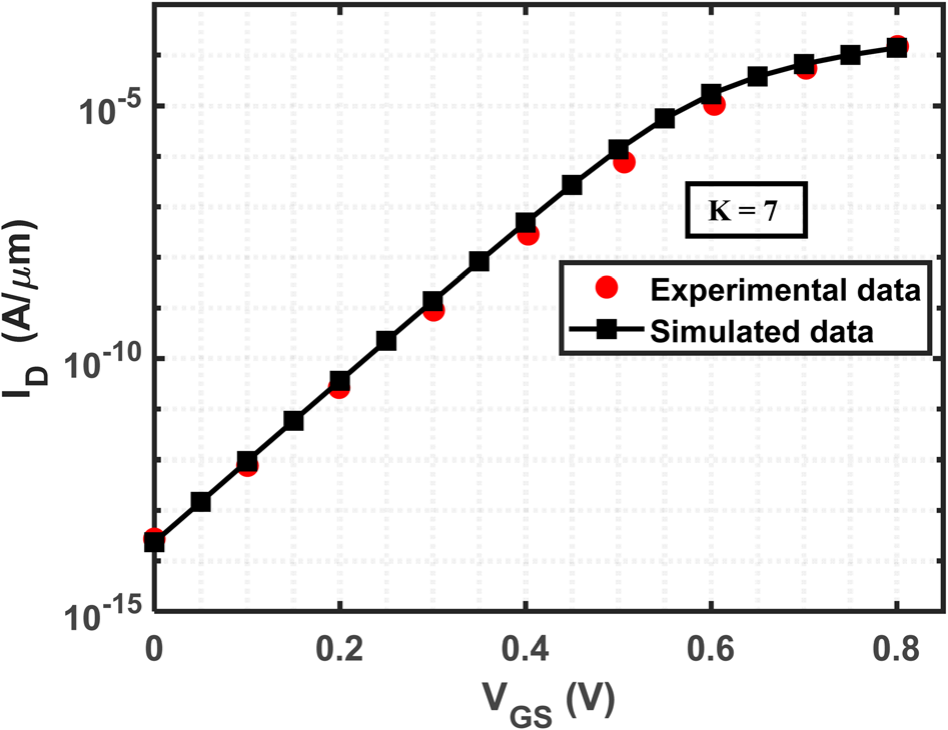
Calibration of the simulation model to replicate the experimental outcomes of a JLFET-based biosensor, as presented in ref. 62. The device specifications include a gate length of 50 nm, cavity length of 15 nm, source/drain length of 20 nm, Si channel diameter of 10 nm, and doping concentration of *N*_D_ = 3 × 10^18^ cm^−3^. The simulation is performed for charge-neutral biomolecules within the cavity region, utilizing a dielectric constant value of 7.

To establish the precision and reliability of the simulation models employed in the research, the biosensor has been calibrated as described in ref. 62. The calibration process involved reproducing the *I*_D_*vs. V*_GS_ characteristics and comparing them with our findings at *V*_DS_ = 1 V. The device parameters were set as follows: gate length, 50 nm; cavity length, 15 nm; source/drain length, 20 nm; Si channel thickness, 10 nm; and doping concentration, *N*_D_ = 3 × 10^18^ cm^−3^. The simulation was conducted for charge-neutral biomolecules within the cavity region with a dielectric constant of 7. Through rigorous analysis, strong consistency was observed between the two datasets, thereby validating the accuracy of the simulation models (Fig. 2).

## 3. Results and discussion

This section provides a detailed comparison and optimization of the seven proposed cavity structures, focusing on various biosensing metrics. The analysis includes *I*_D_*vs. V*_GS_, surface potential, the minimum point of surface potential, change in the minimum point of surface potential, percentage change in the minimum point of surface potential, threshold voltage, change in threshold voltage, percentage change in threshold voltage, *I*_on–off_ ratio, and sensitivity. Based on these metrics, three best-performing structures are identified. Furthermore, the occupancy of the cavities in the best three structures is varied from 50% to 80% of the total channel length to determine the optimal configurations. In addition, the impacts of the gate work function, doping concentration, and gate length are examined to provide comprehensive insights into the factors influencing device performance.

### 3.1. Variation of drain current for different cavity structures

**Fig. 3:**
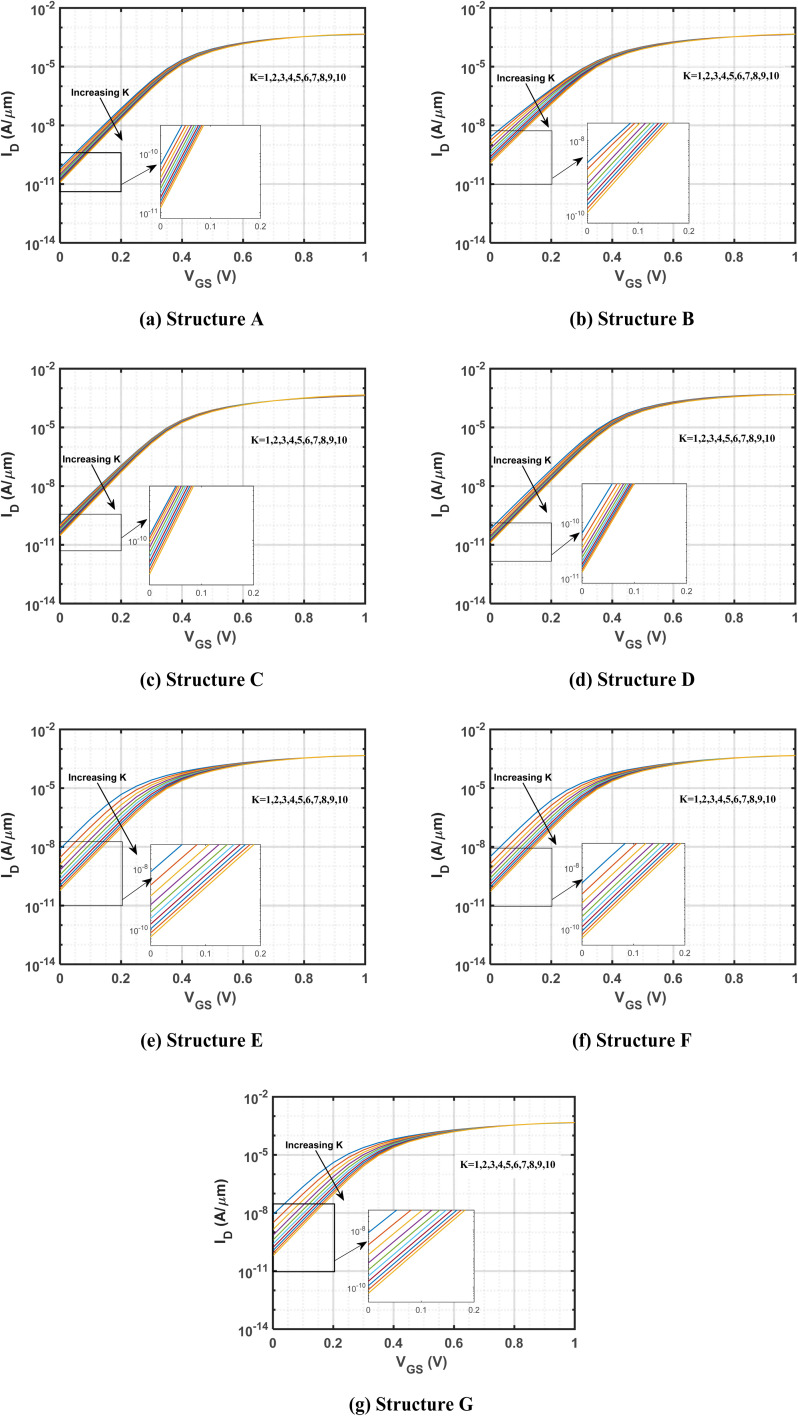
Drain current (*I*_D_) *vs.* gate voltage (*V*_GS_) characteristics for the different cavity structures: (a) structure A, (b) structure B, (c) structure C, (d) structure D, (e) structure E, (f) structure F, and (g) structure G. The device structures have identical parameters, including a drain-to-source voltage of 1 V, a total gate length of 60 nm, a total cavity length of 30 nm and a doping concentration, *N*_D_ = 3 × 10^18^ cm^−3^. The gate voltage is varied from 0 to 1 V for all the structures.

The drain current *vs.* gate voltage characteristics of DG-JLFET-based biosensors with different cavity structures are shown in Fig. 3. The presence of cavity regions in these biosensors plays a pivotal role in modulating carrier transport and electrostatic interactions, thereby significantly influencing drain current behavior. Introducing charge-neutral biomolecules into the cavity alters the device's electrostatic properties by changing the gate capacitance, redistributing the electric field, and affecting channel depletion, all contributing to variations in drain current. A fundamental effect of biomolecule accumulation within the cavity is the increase in total gate capacitance, which results from the higher dielectric constant (*K* > 1) of these biomolecules compared to air (*K* = 1). As the dielectric constant of the cavity region increases, the vertical electric field between the gate and the channel is enhanced, thereby strengthening gate-channel coupling. This improved electrostatic control facilitates more effective carrier depletion, leading to a reduction in the off-state current.^63^ The results reveal a consistent inverse correlation between the dielectric constant and off-state current across all investigated cavity structures, namely A, B, C, D, E, F, and G, as shown in Fig. 3.

The analysis conducted in this study indicates that structure D exhibits the lowest off-state current among all structures for any *K* value ranging from 1 to 10. However, structure C shows the lowest variation in off-state current among them. In contrast, structures E, F, and G show significant variation in off-state current when biomolecules are present in cavities at different positions in the top and bottom oxide regions. Comparing structures C and E, the cavity in structure C is situated on the source side in both the upper and lower sections, whereas in structure E, the cavity is positioned on opposite sides. This configuration in structure E results in a higher degree of variation in the off-state current. Specifically, the cavity spanning the entire gate length by alternating between the top and bottom sections leads to greater variability in the off-state current as the dielectric constant varies within the cavity region, owing to the improved gate controllability.

### 3.2. Variation of surface potential for different cavity structures

**Fig. 4:**
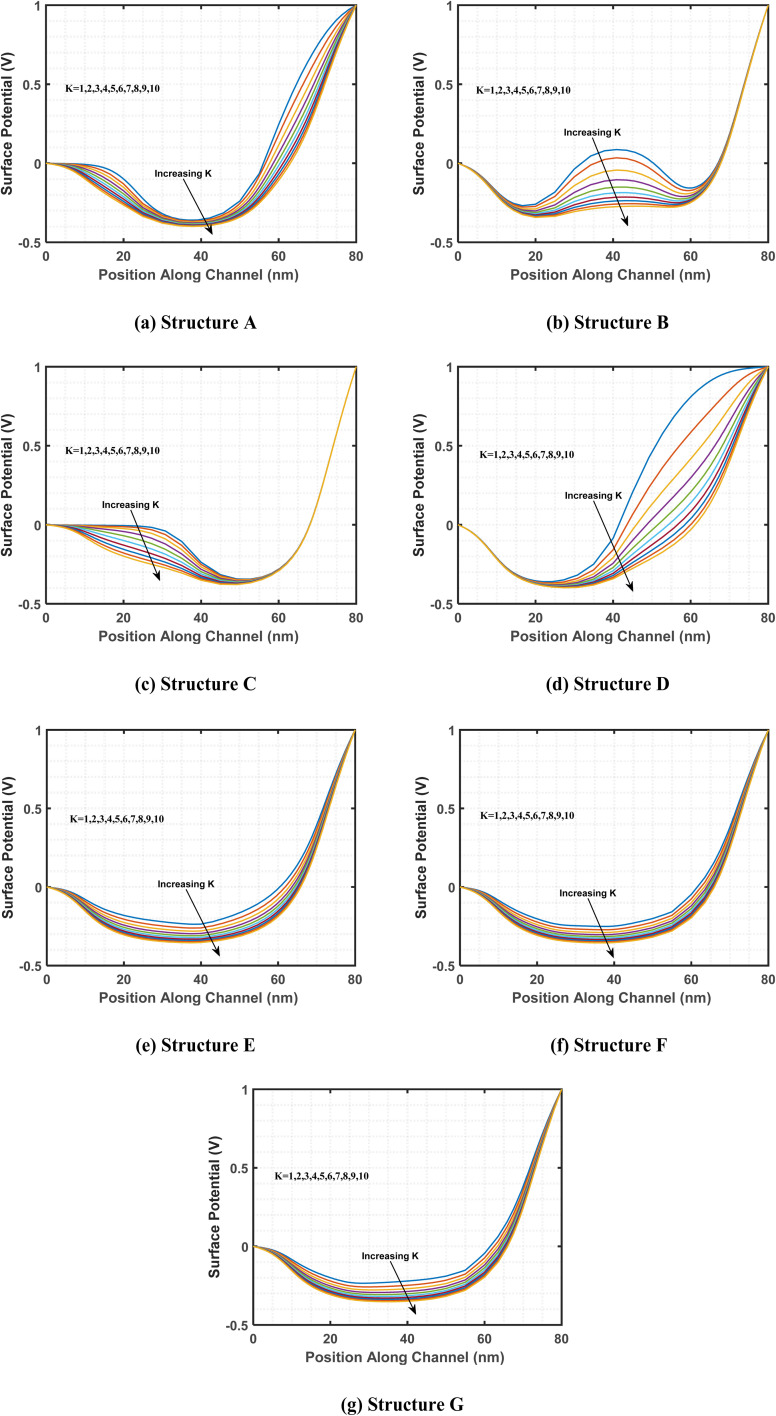
Surface potential across the channel length for various biomolecules with different dielectric constant values, utilizing the following cavity structures: (a) structure A, (b) structure B, (c) structure C, (d) structure D, (e) structure E, (f) structure F, and (g) structure G. To ensure comparability, the devices are designed with identical parameters, including a drain-to-source voltage of 1 V, gate-to-source voltage of 0 V, a total gate length of 60 nm, a total cavity length of 30 nm and a doping concentration of *N*_D_ = 3 × 10^18^ cm^−3^.

This study examines the variations in surface potential along the biosensor channel resulting from changes in the dielectric constant of charge-neutral biomolecules. The objective is to analyze how variations in dielectric properties influence the electrostatic characteristics of biosensors in the presence of biomolecules. Fig. 4 presents a comparison of the surface potential distribution along the channel length for different cavity structures incorporating charge-neutral biomolecules. As previously discussed in Fig. 3, the presence of biomolecules in the cavity region leads to an increase in effective gate capacitance, enhancing gate-channel coupling. This stronger electrostatic interaction results in a reduction in surface potential.^64^ This trend is consistently observed across all examined cavity structures, including A, B, C, D, E, F, and G, highlighting the influence of biomolecule-induced dielectric modulation on the surface potential distribution within the biosensor channel.

With the introduction of biomolecules in the cavity region, the surface potential shows significant fluctuations due to their higher dielectric constant values. Specifically, structure A features cavities on both the left and right sides, while structure E has cavities that span the entire length of the channel, with half positioned at the top and the other half at the bottom of the channel region. As the dielectric constant values increase, these cavity-containing regions exhibit more pronounced variations in surface potential compared to cavity-free regions. This behavior is similarly observed in other structures with cavities.

### 3.3. Variation of biosensing metrics with different cavity structures

**Fig. 5:**
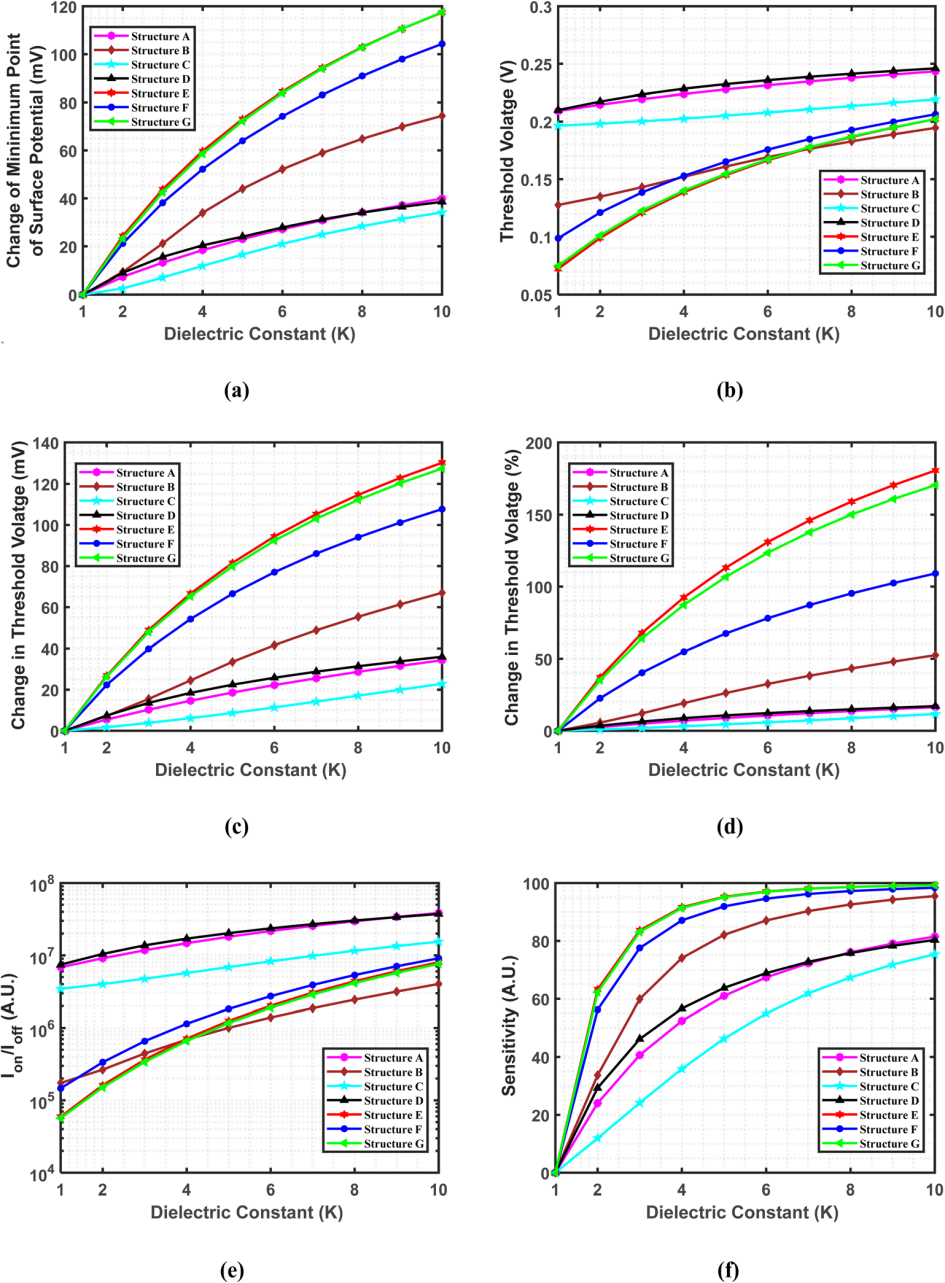
Variation of biosensing metrics with different cavity structures. The impact of charge-neutral biomolecules is analyzed for (a) the change in the minimum point of surface potential, (b) threshold voltage, (c) the change in threshold voltage, (d) the percentage change in threshold voltage, (e) *I*_on–off_ ratio, and (f) sensitivity, all as functions of the dielectric constant. To ensure comparability, the devices are designed with identical parameters, including a drain-to-source voltage of 1 V, a total gate length of 60 nm, a total cavity length of 30 nm, and a doping concentration of *N*_D_ = 3 × 10^18^ cm^−3^.

Fig. 5a presents a comparative analysis of the change in the minimum point of surface potential as a function of the dielectric constant for various cavity structures. As the dielectric constant increases, the minimum surface potential exhibits a consistent downward trend, reflecting the anticipated improvement in gate controllability due to elevated gate capacitance, which in turn reduces the surface potential.^65^ Consequently, across all structures, the change in the minimum point of surface potential increases with the dielectric constant. When comparing structure E with structure C, a significant difference is observed in the change in the minimum point of surface potential as the dielectric constant increases. This behavior is attributed to the stronger electric field gradients generated by the cavities, which span the entire gate length and alternate between the top and bottom sections of the channel. The asymmetric cavity configuration in structure E generates a pronounced electric field gradient across the channel, leading to larger shifts in surface potential as the dielectric constant varies. In contrast, structure C, with both cavities positioned at the source side, experiences a more localized electric field modulation, resulting in smaller variations in the minimum surface potential as the dielectric constant increases. For instance, structure E shows a minimum surface potential of −237.2 mV when the cavity is filled with air, which decreases substantially to −331.56 mV when filled with gluten, leading to a change of 94.36 mV. In comparison, structure C exhibits a more moderate response, with a minimum surface potential of −344.35 mV in the presence of air, which decreases to −369.30 mV when gluten is introduced, resulting in a much smaller change of 24.95 mV. The observation implies that structure E is more influenced by the presence of biomolecules within the cavity, showing a greater change in the minimum point of surface potential.

Fig. 5b shows the variation in threshold voltage with respect to the dielectric constant of charge-neutral biomolecules. An increase in the dielectric constant leads to a rise in threshold voltage (determined by the constant-current method), as the reduction in surface potential enhances carrier depletion within the channel.^66^ As a result, a higher gate voltage is required to induce conduction, causing an overall increase in threshold voltage across all structures. Analysis of structures D and E across all *K* values from 1 to 10 shows that structure D shows the highest threshold voltage. Conversely, structure E shows the lowest threshold voltage for *K* values from 1 to 7. Beyond *K* = 7, structure B exhibits the lowest threshold voltage. The highest threshold voltage in structure D is due to stronger carrier depletion near the source-channel junction, which results in a larger depletion region and a higher potential barrier. This enhanced depletion requires a greater gate voltage to overcome the barrier and initiate conduction. On the other hand, with cavities positioned at both the source and drain sides, structure E experiences reduced carrier depletion across the channel. The more evenly distributed depletion results in a lower potential barrier, thus requiring a lower gate voltage to overcome the depletion and leading to a lower threshold voltage. For example, biomolecules with lower dielectric constants, such as streptavidin and biotin, yield threshold voltages of 0.2180 V and 0.2214 V, respectively, in structure D. In contrast, higher-dielectric constant biomolecules like keratin result in threshold voltages ranging from 0.2414 V to 0.2460 V. Meanwhile, in structure E, the threshold voltages for streptavidin and biotin are significantly lower, measured at 0.1013 V and 0.1133 V, respectively, while keratin shows a threshold voltage of 0.1867–0.2023 V.

Fig. 5c shows a comparative analysis of the changes in threshold voltage across various cavity structures with the dielectric constant, highlighting the impact of cavity structure on device electrical performance. The data, calculated using eqn (2), show an increase in the change in threshold voltage for all structures. This increase is linked to a rise in the dielectric constant within the cavity region, which subsequently lowers the minimum point of surface potential.^63^ This phenomenon persists across a wide range of *K* values. As shown in Fig. 5a, structure E exhibits the largest change in the minimum point of surface potential, resulting in the most significant change in threshold voltage. For instance, with biomolecules like keratin and SARS-COV-2, there is a noticeable variation in the minimum surface potential between structure E and structure C, as shown in Fig. 5a. This difference leads to a significant change in threshold voltage for structure E compared to structure C.

Fig. 5d presents the percentage change in threshold voltage for different cavity structures, calculated using eqn (3). As the dielectric constant increases, the threshold voltage also rises, resulting in a corresponding increase in the percentage change for all examined cavity structures—namely structures A, B, C, D, E, F, and G.

Among all the structures, structure C demonstrates the lowest percentage change in threshold voltage, while structure E shows the highest. For example, with biomolecules like gluten, structure C shows a percentage change in threshold voltage of only 7.23%, whereas it rises significantly to 146.07% for structure E.

Fig. 5e highlights the importance of the *I*_on–off_ ratio, which measures the variation between on and off-state currents, in determining the detectability of biological sensors. This metric is calculated using eqn (4). As observed in Fig. 3, while the on-state current remains relatively constant, the off-state current exhibits significant variations with changes in the dielectric constant. Notably, an increase in *K* leads to a decrease in off-state current, consequently enhancing the *I*_on–off_ ratio. This trend is observed across various structures, namely A, B, C, D, E, F, and G. Upon analyzing the variation in off-state current across different structures, a significant difference in the *I*_on–off_ ratio is observed between structures B, D, and G. Structure D shows the highest *I*_on–off_ ratio across the range of *K* values from 1 to 10, due to its lowest off-state current. In contrast, structure G shows the lowest *I*_on–off_ ratio for *K* = 1 to 4, while structure B exhibits the lowest values beyond *K* = 4. As previously discussed, the cavities positioned on the same side in structure D induce stronger carrier depletion, which enhances the suppression of off-state current. This enhanced depletion results in a higher *I*_on–off_ ratio for structure D. Conversely, structures E and G, which exhibit reduced carrier depletion, result in less effective suppression of off-state current, thereby contributing to a lower *I*_on–off_ ratio in these structures. Despite performing better in terms of the change and percentage change in threshold voltage, structure G shows the lowest *I*_on–off_ ratio for *K* values up to 4. Importantly, structure D not only demonstrates the highest *I*_on–off_ ratio but also achieves the highest threshold voltage underscoring its exceptional performance across these critical biosensing metrics. For instance, in structure D, the *I*_on–off_ ratio reaches 1.078 × 10^7^ for streptavidin and 1.207 × 10^7^ for protein, the biomolecules with lower dielectric constants, while it increases to 3.03 × 10^7^–3.73 × 10^7^ for molecules with higher dielectric constants, such as keratin.

Fig. 5f illustrates how the device's sensitivity changes when biomolecules with different *K* values are introduced into the cavity region for various cavity structures, as calculated using eqn (1). As the dielectric constant increases, the off-state current decreases, thereby increasing the sensitivity of all structures. Notably, structures E and G show the most significant changes in off-state current with increasing *K* values, resulting in higher sensitivity compared to other structures. In contrast, structure C shows minimal changes in off-state current, leading to lower sensitivity. The observed differences in sensitivity are due to the varying cavity configurations across different structures. When the cavities span the entire channel length, alternating between the upper and lower sections, the initial gate control is weaker when no biomolecule is present. However, when charge-neutral biomolecules are introduced into these structures, their dielectric properties significantly enhance the electric field gradient, which improves gate control. This results in larger variations in off-state current, leading to higher sensitivity. In contrast, when both cavities are positioned on the same side, the electric field gradient is weaker, causing smaller variations in off-state current and, consequently, lower sensitivity as dielectric constant changes. Thus, structures E and G are particularly responsive to charge-neutral biomolecul2es, making them suitable candidates for DG-JLFET-based sensors. Notably, structure E demonstrates a sensitivity of 66.22% for streptavidin and sensitivities ranging from 98.63% to 99.25% for keratin.

Table 4 offers a detailed ranking of various structures based on multiple sensing metrics, including threshold voltage, change in threshold voltage, percentage change in threshold voltage, *I*_on–off_ ratio, sensitivity, and change in the minimum point of surface potential, as the dielectric constant of the cavity region varies.

**Table 4:** Comparison and ranking of different cavity configurations based on various biosensing metrics

Structure	Threshold voltage	Change in threshold voltage	Change in threshold voltage	*I*_on–off_ ratio	Sensitivity	Change in the minimum point of surface potential
A	2	6	6	2	6 up to *K* = 8, then 5	6 up to *K* = 8, then 5
B	4 up to *K* = 4, then 5 up to *K* = 7, then 7	4	4	5 up to *K* = 4, then 7	4	4
C	3	7	7	3	7	7
D	1	5	5	1	5 up to *K* = 8, then 6	5 up to *K* = 8, then 6
E	7 up to *K* = 7, then 6	1	1	6 up to *K* = 4, then 5	1	1
F	5 up to *K* = 4, then 4	3	3	4	3	3
G	6 up to *K* = 7, then 5	2	2	7 up to *K* = 4, then 6	2	2

No single structure excels in all categories. Structure D ranks highest in both the threshold voltage and *I*_on–off_ ratio metrics, whereas structure E shows the highest change and percentage change in threshold voltage, the highest sensitivity, and the largest change in the minimum point of the surface potential. Despite its strong performance across most metrics, structure E has ranked poorly in threshold voltage (7th up to *K* = 7, then 6th) and *I*_on–off_ ratio (6th up to *K* = 4, then 5th). However, when sensitivity is prioritized as the key biosensing metric, structures E, F, and G outperform the other structures. These three structures are, therefore, selected for further investigations in this study.

Table 5 shows the performance of structures D and E for practical biomolecules present in the cavity region. It reveals that structure D maintains a superior *I*_on–off_ ratio, while structure E shows the highest sensitivity. These findings emphasize the unique advantages of each structure, facilitating the selection of appropriate cavity structures tailored to specific biosensing metrics.

**Table 5:** The sensitivity and *I*_on–off_ ratio of practical biomolecules in best-performing cavity structures

Biomolecules name	*I*_on–off_ ratio		Sensitivity	
	Structure D	Structure E	Structure D	Structure E
Streptavidin	1.078 × 10^7^	1.76 × 10^5^	31.36%	66.22%
Biotin	1.25 × 10^7^	2.755 × 10^5^	40.90%	78.31%
APTES	1.558 × 10^7^	3.419 × 10^5^	52.70%	88.98%
Protein	1.207 × 10^7^	2.478 × 10^5^	38.79%	75.87%
Gluten	2.693 × 10^7^	3.056 × 10^6^	72.73%	98.04%
Zein	2.029 × 10^7^–2.693 × 10^7^	1.249 × 10^6^–3.056 × 10^6^	63.74%–72.73%	95.22%–98.04%
Keratin	3.03 × 10^7^ – 3.73 × 10^7^	4.388 × 10^6^–8.016 × 10^6^	75.79%–80.32%	98.63%–99.25%
SARS-COV-2	1.70 × 10^7^, 3.73 × 10^7^	7.11 × 10^5^, 8.016 × 10^6^	56.66%, 80.32%	91.61%, 99.25%

### 3.4. Investigation of the biosensors' performance dependence on channel occupancy

**Fig. 6:**
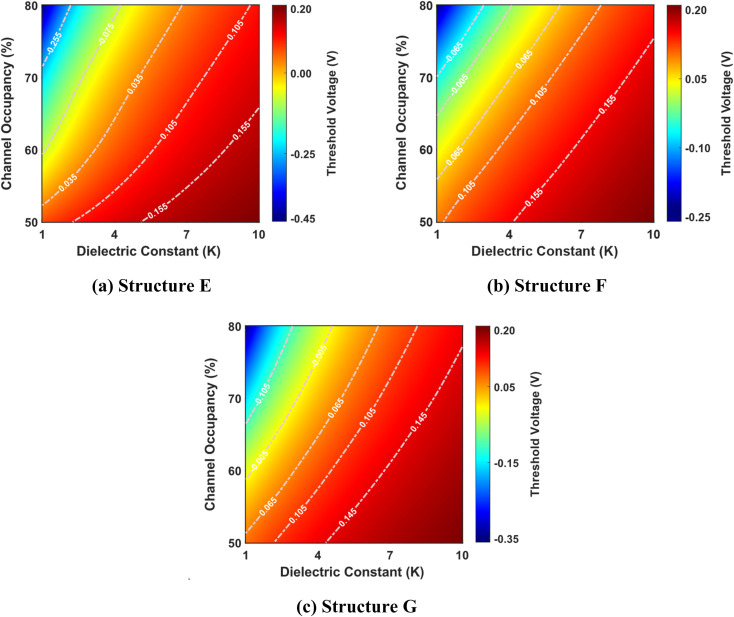
Threshold voltage as a function of dielectric constant and channel occupancy for three best-performing cavity structures: (a) structure E, (b) structure F, and (c) structure G. The graphs are shown for cavity lengths ranging from 50% to 80% of the total channel length (where the total channel length is 60 nm). The devices have identical parameters, including a drain-to-source voltage of 1 V, a total gate length of 60 nm, a total cavity length of 30 nm, and a doping concentration of *N*_D_ = 3 × 10^18^ cm^−3^.

Fig. 6 presents the intricate interplay between the dielectric constant, channel occupancy, and their collective impact on the threshold voltage for structures E, F, and G, with cavity lengths ranging from 50% to 80% of the total channel length. The figure reveals a distinct trend: as the dielectric constant increases for a given channel occupancy, the threshold voltage correspondingly rises. This phenomenon is linked to the corresponding reduction in surface potential, which drives the increase in threshold voltage.

Conversely, the threshold voltage demonstrates a declining trend as channel occupancy increases for a specific dielectric constant. This behavior is rooted in the diminishing effective capacitance between the gate and the channel as the cavity length extends, leading to a weaker gate control over the channel. The nuanced relationship between the dielectric constant and channel occupancy underscores the pivotal role of cavity architecture in defining the electrostatic characteristics of these structures.

**Fig. 7:**
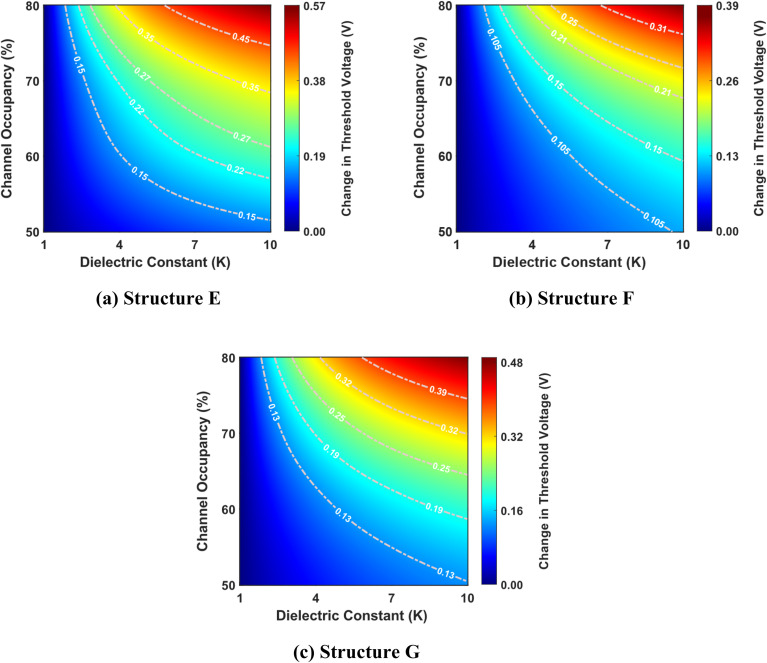
Change in threshold voltage as a function of dielectric constant and channel occupancy for three best-performing cavity structures: (a) structure E, (b) structure F, and (c) structure G. The graphs are shown for cavity lengths ranging from 50% to 80% of the total channel length (where the total channel length is 60 nm). The devices have identical parameters, including a drain-to-source voltage of 1 V, a total gate length of 60 nm, a total cavity length of 30 nm, and a doping concentration of *N*_D_ = 3 × 10^18^ cm^−3^.

Fig. 7 shows the change in threshold voltage as a function of the dielectric constant and channel occupancy for structures E, F, and G. As previously shown in Fig. 5b, the threshold voltage increases as the dielectric constant changes from *K* = 1 to *K* = 10 for a fixed channel occupancy, resulting in a similar trend in the change in threshold voltage. This pattern is mirrored as channel occupancy increases for a fixed dielectric constant, indicating a complementary relationship between these two parameters. Remarkably, structure E exhibits a greater change in threshold voltage compared to structures F and G. This suggests that structure E possesses a higher responsiveness to changes in dielectric constant and channel occupancy, thereby outperforming the other two structures in this regard.

**Fig. 8:**
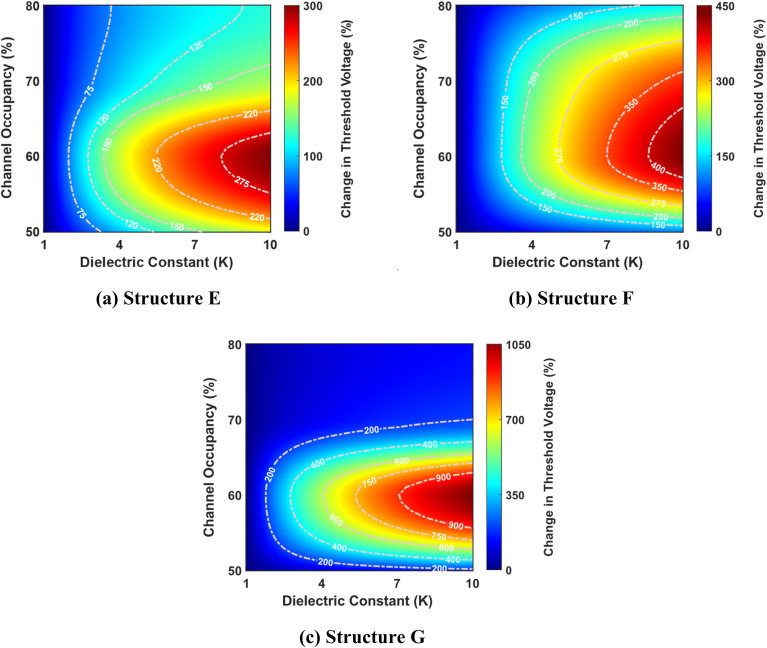
Percentage change in threshold voltage as a function of dielectric constant and channel occupancy for three best-performing cavity structures: (a) structure E, (b) structure F, and (c) structure G. The graphs are shown for cavity lengths ranging from 50% to 80% of the total channel length (where the total channel length is 60 nm). The devices have identical parameters, including a drain-to-source voltage of 1 V, a total gate length of 60 nm, a total cavity length of 30 nm, and a doping concentration of *N*_D_ = 3 × 10^18^ cm^−3^.

Fig. 8 shows the complex interplay between the dielectric constant, channel occupancy, and the resulting percentage change in threshold voltage. It reveals a clear trend of increasing percentage change in threshold voltage change with rising dielectric constant values. This effect is further amplified at higher channel occupancy levels, demonstrating a synergistic relationship between these two parameters. Interestingly, the plot also hints at a non-linear dependency, evidenced by the non-uniform spacing of the contour lines.

Furthermore, the figure unveils an intriguing phenomenon: for a specific dielectric constant, there appears to be a local maximum in the percentage change in threshold voltage as channel occupancy increases. This suggests a complex, non-monotonic relationship between these two factors, potentially indicating an optimal channel occupancy level for maximizing the change in threshold voltage. Among the three structures considered, structure G shows the highest percentage change in threshold voltage, while structure E exhibits the lowest.

**Fig. 9:**
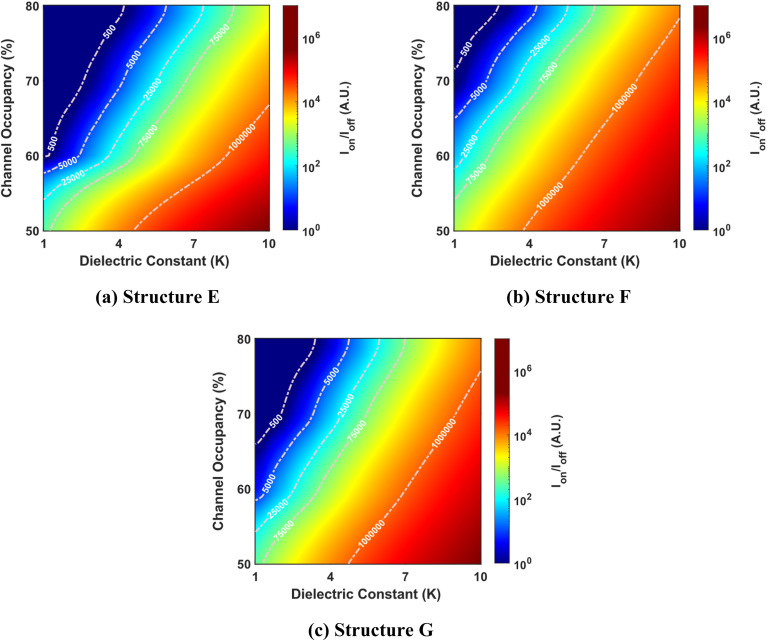
*I*_on–off_ ratio as a function of dielectric constant and channel occupancy for three best-performing cavity structures: (a) structure E, (b) structure F, and (c) structure G. The graphs are shown for cavity lengths ranging from 50% to 80% of the total channel length (where the total channel length is 60 nm). The devices have identical parameters, including a drain-to-source voltage of 1 V, a total gate length of 60 nm, a total cavity length of 30 nm, and a doping concentration of *N*_D_ = 3 × 10^18^ cm^−3^.

Fig. 9 provides an insightful depiction of how the *I*_on–off_ ratio varies with the dielectric constant and channel occupancy for structures E, F, and G. Notably, an increase in the dielectric constant, while maintaining a constant channel occupancy, results in an increase in the threshold voltage. This increase in threshold voltage reduces the off-state current, which enhances the *I*_on–off_ ratio across all structures.

In contrast, as illustrated in Fig. 6, an increase in channel occupancy results in a decrease in the threshold voltage for a specific dielectric constant. This reduction in threshold voltage leads to an increased off-state current, which subsequently diminishes the *I*_on–off_ ratio across all three structures. The inverse relationship between channel occupancy and the *I*_on–off_ ratio is consistently observed in structures E, F, and G, underscoring the importance of balancing these parameters to optimize device performance. Among the three structures analyzed, structure F exhibits the highest *I*_on–off_ ratio, performing better than the other structures.

**Fig. 10:**
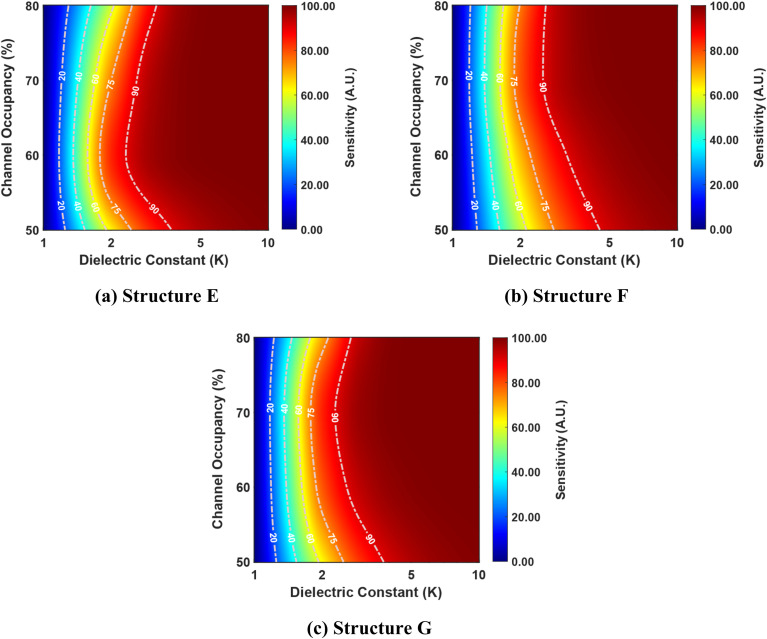
Sensitivity as a function of dielectric constant and channel occupancy for three best-performing cavity structures: (a) structure E, (b) structure F, and (c) structure G. The graphs are shown for cavity lengths ranging from 50% to 80% of the total channel length (where the total channel length is 60 nm). The devices have identical parameters, including a drain-to-source voltage of 1 V, a total gate length of 60 nm, a total cavity length of 30 nm, and a doping concentration of *N*_D_ = 3 × 10^18^ cm^−3^.

Fig. 10 presents the correlation between the dielectric constant, channel occupancy, and sensitivity for structures E, F, and G. It reveals a clear trend where sensitivity increases with higher dielectric constant values, while channel occupancy remains constant, as discussed earlier.

However, for low dielectric constant values, sensitivity remains relatively invariant with respect to changes in channel occupancy, indicating a lack of dependence between these parameters. Interestingly, as the dielectric constant value increases, sensitivity values initially increase with rising channel occupancy up to a specific threshold, beyond which they decrease. This behavior suggests a non-linear relationship between sensitivity and channel occupancy, potentially indicative of an optimal channel occupancy level for maximizing sensitivity. As the dielectric constant value exceeds 5, the channel dependence of the sensitivity vanishes.

Additional investigations into the impact of gate work function, doping concentration, and gate length on biosensing performance have been conducted to provide a more comprehensive analysis. A detailed discussion of these results is available in the ESI,† ensuring accessibility while keeping the primary focus on key findings.

## 4. Summary, conclusion, and outlook

In summary, this study has evaluated the performance of different cavity structures in DG-JLFETs for enhanced biomolecule detection. The findings indicate significant improvements in biosensing performance across various cavity configurations with respect to key sensing metrics, such as threshold voltage, change in threshold voltage, the percentage change in threshold voltage, *I*_on–off_ ratio, sensitivity, and change in the minimum point of surface potential. The analysis reveals that no single structure has outperformed the others in all the biosensing metrics. For instance, structure D, with its symmetrical cavity architecture, has achieved the highest threshold voltage and *I*_on–off_ ratio for *K* values ranging from 1 to 10. At *K* = 10, it has shown a threshold voltage of 0.2459 V and an *I*_on–off_ ratio of 3.73 × 10^7^. Meanwhile, structure E has surpassed the other structures in the remaining biosensing metrics. For example, for *K* = 10, structure E has yielded a change in threshold voltage of 130.24 mV, a percentage change in threshold voltage of 180.72%, a sensitivity of 99.25%, and a change in the minimum point of the surface potential of 117.39 mV. This superiority of structure E has been maintained over a wide range of dielectric constant values. To further optimize biosensing performance, the cavity occupancy in the three best-performing structures was varied between 50% and 80% of the total channel length. The results have shown that structure E exhibits the highest change in threshold voltage, whereas structure G achieves the highest percentage change in threshold voltage. However, structure F outperforms the others in terms of the *I*_on–off_ ratio, indicating enhanced switching behavior in this evaluation.

The proposed DG-JLFET-based biosensors offer significant practical advantages, particularly in real-time, label-free biomolecule detection. Their compact design and seamless integration into lab-on-a-chip systems make them highly suitable for point-of-care diagnostics, environmental monitoring, and food safety applications. Moreover, the uniform doping concentration in JLFETs simplifies fabrication, improving scalability and compatibility with CMOS technology. In addition, their strong electrostatic control enables greater detection sensitivity, even for low-concentration biomolecules. However, despite these advantages, certain fabrication challenges remain. The need for precise lithographic alignment, complex masking, and advanced etching techniques increases production complexity and may raise manufacturing costs. Furthermore, factors such as noise susceptibility, parasitic capacitance, and surface roughness effects can affect sensor stability and detection accuracy, necessitating the development of enhanced noise reduction strategies and optimized signal processing techniques. Addressing these limitations through advancements in fabrication methods and detection algorithms will be crucial for improving the scalability, reliability, and overall performance of these biosensors. With continued innovation, DG-JLFET-based biosensors hold significant potential for next-generation diagnostic and sensing applications.

Regarding the future scope of research, subsequent studies can build upon the conclusions drawn from this study by exploring additional dimensions to enhance biomolecule detection capabilities. One potential area for future investigation is the study of charged biomolecules and their interactions with cavity structures in DM-DG JLFET biosensors, as well as the impact of charge distribution and density on device sensitivity. Additionally, studying the effects of biomolecule arrangement and cavity filling, such as partial cavity filling, nonuniform biomolecule arrangement, and the influence of steric hindrance on biomolecule binding, could provide valuable insights. Furthermore, examining the multi-gate work function in the cavity region of DG-JLFET biosensors may offer additional design considerations for creating highly sensitive and efficient biosensors. Lastly, investigating the effects of temperature and pH on the performance of DG-JLFET biosensors and developing strategies to enhance their resilience to these fluctuations would be beneficial for practical applications.

## Data availability

The data that support the findings of this study are available from the corresponding author upon reasonable request.

## Conflicts of interest

The authors declare no conflict of interest.

## Supplementary Material

NA-OLF-D4NA00928B-s001
