# Supplementary material for: Designing the cavity architecture in double gate junctionless field effect transistors for enhanced biomolecule detection
Source: Nanoscale Adv. 2025 Apr 25;7(12):3746–63. doi: 10.1039/d4na00928b (PMC12070517; doi:10.1039/d4na00928b)
Supplement: NA-007-D4NA00928B-s001 [file NA-007-D4NA00928B-s001.pdf]

Supplementary Information (SI) for Nanoscale Advances.
This journal is © The Royal Society of Chemistry 2025

Supplementary Information

Designing the Cavity Architecture in Double Gate Junctionless Field Effect Transistor for Enhanced Biomolecule Detection

Shahriar Khan[1] and Ehsanur Rahman[1*]

[1] Department of Electrical and Electronic Engineering, Bangladesh University of Engineering and Technology, Dhaka,1000, Bangladesh

*Email: ehsaneee@eee.buet.ac.bd

Variation of Biosensing Metrics with Different Cavity Structure

Figure S1 presents a comparative analysis of the minimum point of surface potential as a function of the dielectric constant for various cavity structures. As discussed earlier in **figure 3.3a,** the minimum surface potential exhibits a decreasing trend with the increased dielectric constant values. This trend is consistently observed across structures A, B, C, D, E, F, and G. Notably, structures A and D show the lowest minimum surface potential values across a wide range of dielectric constant values. In contrast, structure G exhibits the highest values for K = 1 to 6, whereas for K values exceeding 6, structure B demonstrates the highest minimum surface potential values.

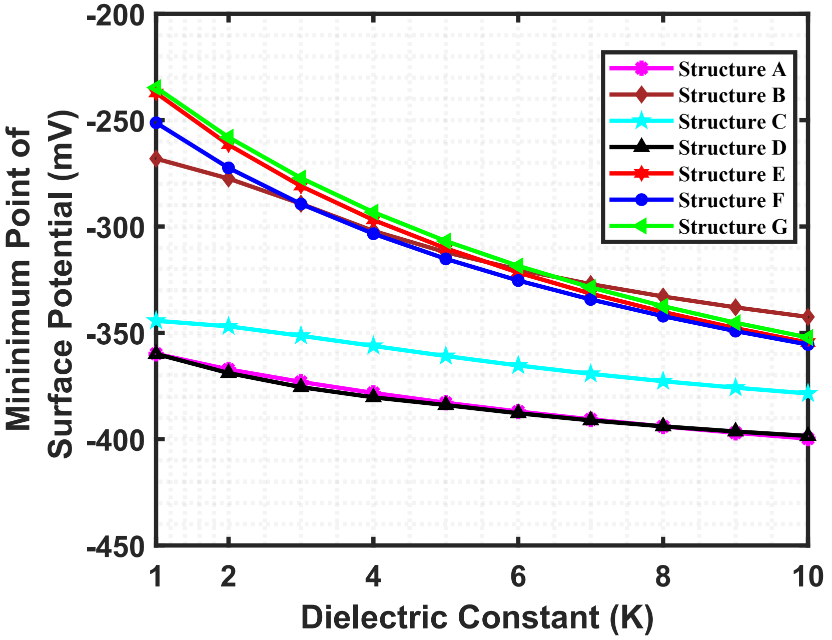

Figure S1: Effect of charge-neutral biomolecules on the minimum point of surface potential as a function of the dielectric constant for various cavity structures. To ensure comparability, the devices are designed with identical parameters, including a drain-to-source voltage of 1 V, a total gate length of 60 nm, a total cavity length of 30 nm, and a doping concentration of $N_D = 3 \times 10^{18}$ cm^{-3}.

The comparative analysis presented in figure S2 shows the percentage change in the minimum point of surface potential for various cavity structures. Notably, the observed trend in

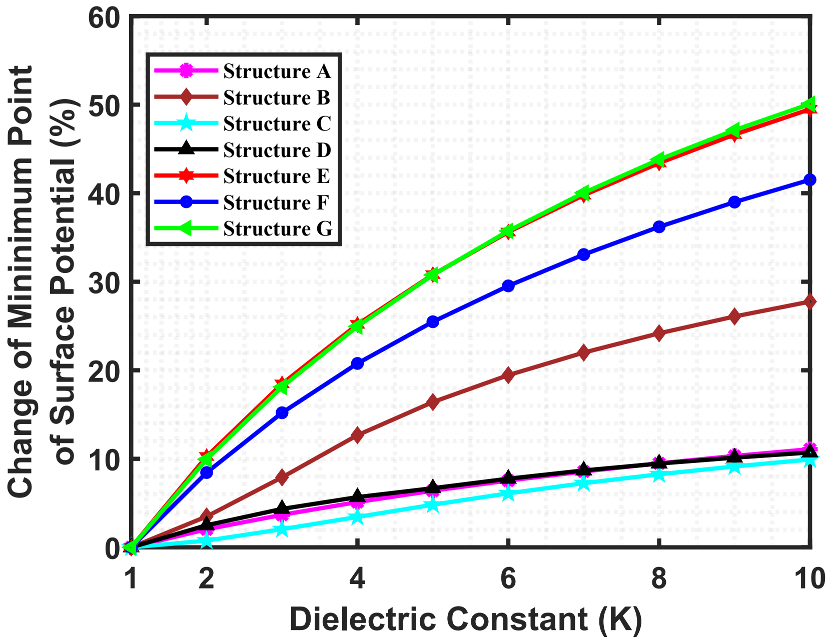

Figure S2: Effect of charge-neutral biomolecules on the percentage change of the minimum point of surface potential as a function of the dielectric constant for various cavity structures. To ensure comparability, the devices are designed with identical parameters, including a drain-to-source voltage of 1 V, a total gate length of 60 nm, a total cavity length of 30 nm, and a doping concentration of $N_D = 3 \times 10^{18}$ cm^{-3}.

the incremental shift of the change in the minimum point of surface potential, as depicted in **figure 3.3b**, is reflected in the percentage change for all structures. Upon examining the dielectric constant values ranging from 1 to 10, it becomes evident that structure C shows the most minimal percentage change in the minimum point of surface potential. In contrast, structures E and G show the most significant percentage changes.

The Impact of Gate Work Function on Biosensing Metrics

Figure S3 shows the drain current vs. gate voltage characteristics for two different values, (φ = 4.88 eV and 5.25 eV) of the gate work function, illustrating that a higher gate work function (φ = 5.25 eV) leads to a reduced off-state current. This observation aligns with the principles of thermionic emission theory, which posits that the current density decreases exponentially as the barrier height increases [1].

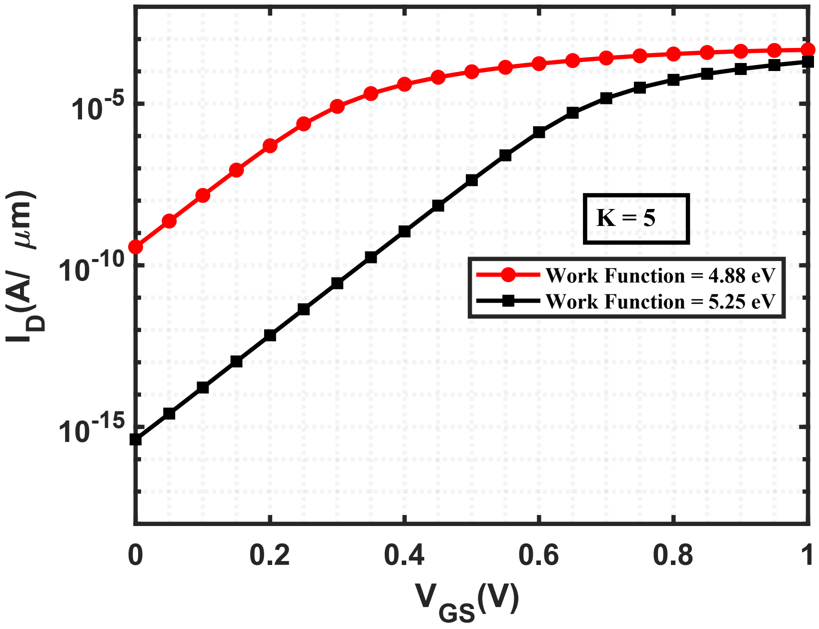

Figure S3: Drain current vs. gate voltage characteristics for two different work functions (φ = 4.88 eV and 5.25 eV) of the gate electrode. The graphs are presented for structure E with a dielectric constant of 5. The devices have identical parameters to ensure comparability, including a drain-to-source voltage of 1 V, a total gate length of 60 nm, a total cavity length of 30 nm, and a doping concentration of $N_D = 3 \times 10^{18}$ cm^{-3}.

Consequently, a greater difference between the work function of the gate material and the semiconductor contributes to a substantial reduction in the off-state current. This phenomenon explains the observed decline at higher work function values.

Figure S4 shows the threshold voltage for two different work function values (φ = 4.88 eV and 5.25 eV), displaying a similar trend as previously discussed. A higher work function results

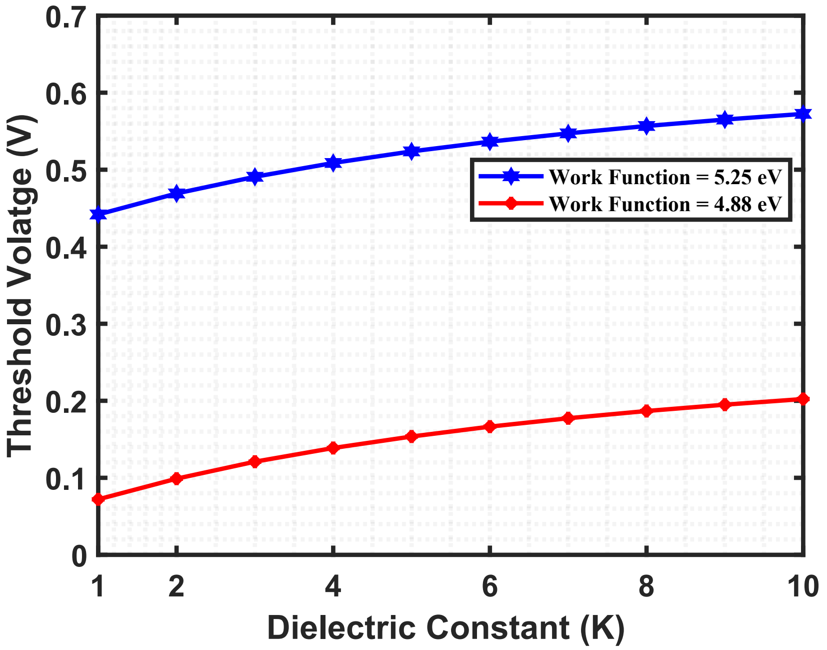

Figure S4: Impact of charge-neutral biomolecules on threshold voltage with varying dielectric constant within the cavity region for structure E, considering two different work functions (φ = 4.88 eV and 5.25 eV) of the gate electrode. To ensure comparability, the devices have identical parameters, including a drain-to-source voltage of 1 V, a total gate length of 60 nm, a total cavity length of 30 nm, and a doping concentration of $N_D = 3 \times 10^{18}$ cm^{-3}.

in a greater energy barrier for electrons, increasing the barrier height, and hindering the formation of a conducting channel at the surface. As a result, a more positive gate voltage is required for channel formation, which increases the threshold voltage [2].

Figure S5 shows the change in threshold voltage for two different work function values, φ = 4.88 eV and 5.25 eV. As discussed earlier, an increasing trend in the change in the threshold voltage was observed with respect to the dielectric constant for both work function values.

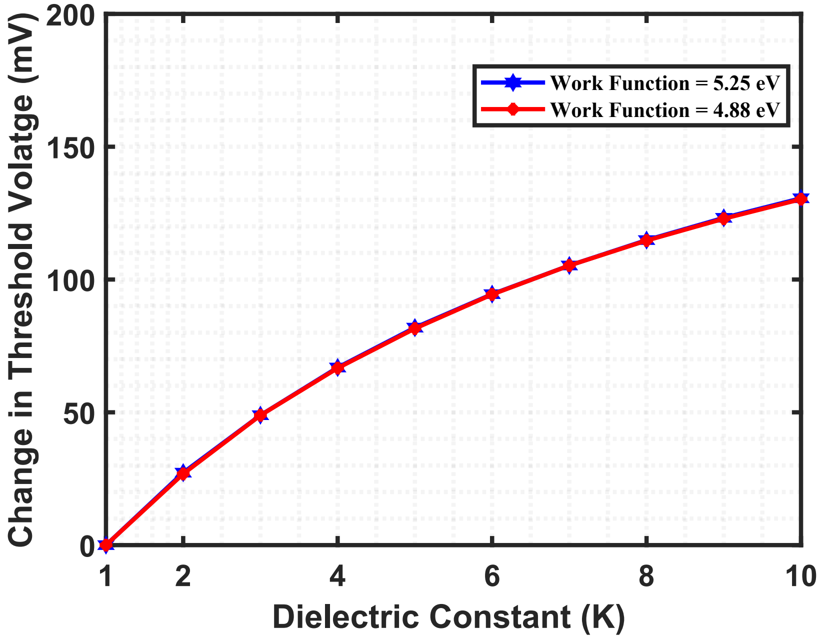

Figure S5: Impact of charge-neutral biomolecules on the change in threshold voltage with varying dielectric constant within the cavity region for structure E, considering two different work functions (φ = 4.88 eV and 5.25 eV) of the gate electrode. To ensure comparability, the devices have identical parameters, including a drain-to-source voltage of 1 V, a total gate length of 60 nm, a total cavity length of 30 nm, and a doping concentration of $N_D = 3 \times 10^{18}$ cm^{-3}.

The analysis reveals that the change in threshold voltage is identical for both work function values as the dielectric constant is varied from 1 to 10, allowing for the strategic optimization of the gate work function to improve other biosensing metrics without adversely affecting this particular parameter.

Figure S6 presents the percentage change in threshold voltage for structure E, considering work functions of 4.88 eV and 5.25 eV, calculated using equation 2.3. As the threshold voltage increases with the dielectric constant, the figure indicates an increasing trend for both the work functions, as explained earlier. However, the percentage change in the threshold voltage with respect to the dielectric constant is greater for lower work function values than for higher work function values. Previous analysis indicated that higher work function values resulted in a higher

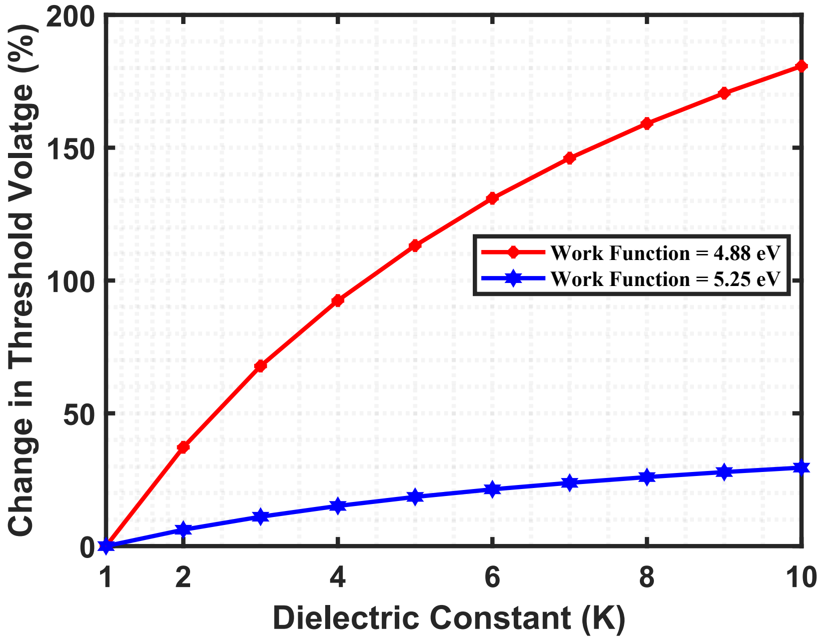

Figure S6: Impact of charge-neutral biomolecules on the percentage change in threshold voltage with varying dielectric constant within the cavity region for structure E, considering two different work functions (φ = 4.88 eV and 5.25 eV) of the gate electrode. To ensure comparability, the devices have identical parameters, including a drain-to-source voltage of 1 V, a total gate length of 60 nm, a total cavity length of 30 nm, and a doping concentration of $N_D = 3 \times 10^{18}$ cm^{-3}.

threshold voltage at K = 1, supporting the observation that the threshold voltage experiences a more significant increase at lower work functions as the dielectric constant increases.

Figure S7 presents a comparative analysis of the Ion-off ratio as a function of the dielectric constant for two different work function values (φ = 4.88 eV and 5.25 eV). As discussed earlier, a similar trend in the Ion-off ratio is observed for both work function values as the dielectric constant varies. However, a higher work function increases the energy barrier between the drain and the source terminals, reducing the number of carriers and lowering the off-state current while maintaining a relatively constant on-state current, as shown in figure S3. This behavior is consistent across all the dielectric constant values in the cavity region, resulting in a higher Ion-off ratio for a higher gate work function.

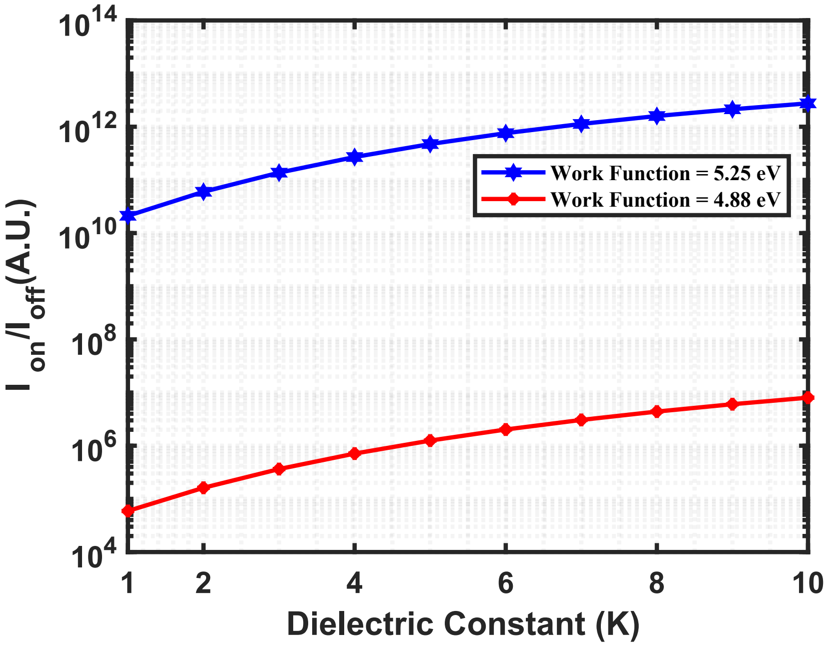

Figure S7: Impact of charge-neutral biomolecules on Ion-off ratio with varying dielectric constant within the cavity region for structure E, considering two different work functions (φ = 4.88 eV and 5.25 eV) of the gate electrode. To ensure comparability, the devices have identical parameters, including a drain-to-source voltage of 1 V, a total gate length of 60 nm, a total cavity length of 30 nm, and a doping concentration of $N_D = 3 \times 10^{18}$ cm^{-3}.

Figure S8 presents a comparative analysis of sensitivity as a function of the dielectric constant for two different work function values (φ = 4.88 eV and 5.25 eV). As previously explained, an increasing trend is observed in sensitivity with the dielectric constant. Upon analyzing the sensitivity for the two work function values, it is noted that within the range of K = 2 to K = 6, the sensitivity is slightly greater for the gate with a higher work function. Outside this range, the sensitivities of the two work function values are almost the same.

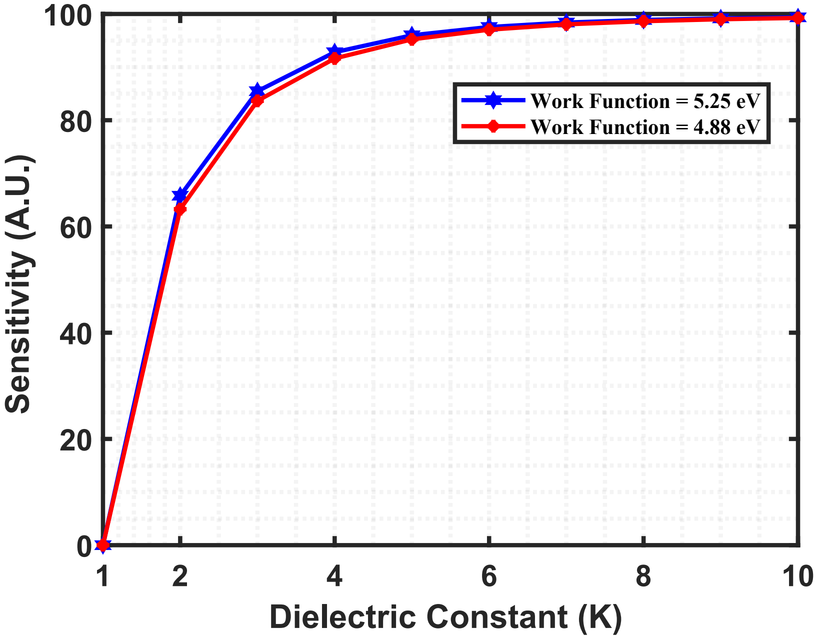

Figure S8: Impact of charge-neutral biomolecules on sensitivity with varying dielectric constant within the cavity region for structure E, considering two different work functions (φ = 4.88 eV and 5.25 eV) of the gate electrode. The devices have identical parameters to ensure comparability, including a drain-to-source voltage of 1 V, a total gate length of 60 nm, a total cavity length of 30 nm, and a doping concentration of $N_D = 3 \times 10^{18}$ cm^{-3}.

The Impact of Doping Concentration on Biosensing Metrics

Figure S9 shows the drain current vs. gate voltage characteristics for two different doping concentrations in the channel region of structure E. A higher doping concentration significantly increases the off-state current. This occurs because increasing the doping concentration in the channel leads to more free carriers, resulting in an increased leakage current through the channel, even in the off-state [3]. This explains the increased off-state current at higher doping concentrations.

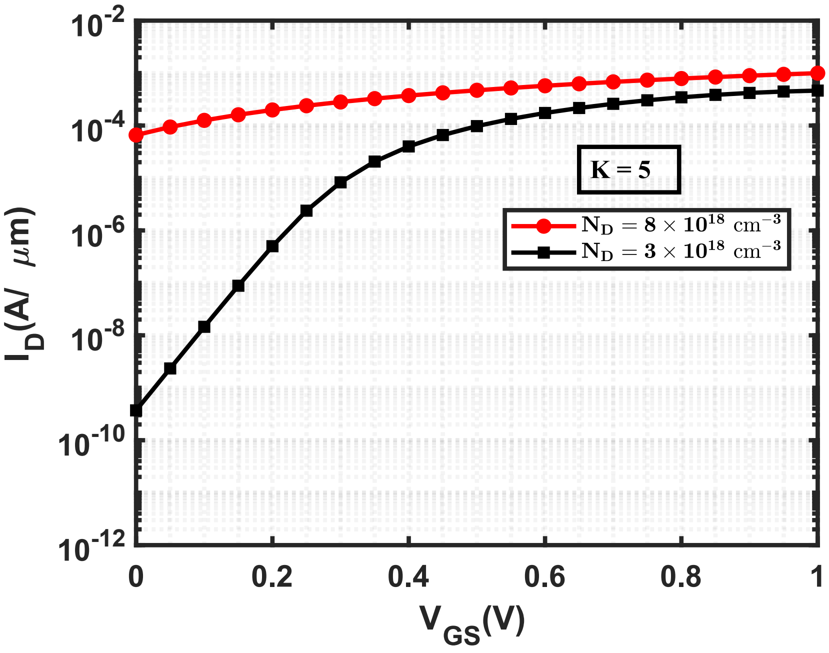

Figure S9: Drain current vs. gate voltage characteristics for two different doping concentrations (N_D = 3 × 10^{18} cm^{-3} and 8 × 10^{18} cm^{-3}). The graphs are shown for structure E with a dielectric constant value of 5. To ensure comparability, the devices have identical parameters, including a drain-to-source voltage of 1 V, a total gate length of 60 nm, a total cavity length of 30 nm, and the work function of a gate electrode, φ = 4.88 eV.

Figure S10 shows the threshold voltage of structure E for two different doping concentrations, N_D = 3 × 10^{18} cm^{-3} and 8 × 10^{18} cm^{-3}. The general trend of the threshold voltage, obtained using the constant current method, with respect to the dielectric constant of the cavity region follows a similar pattern to what was previously explained.

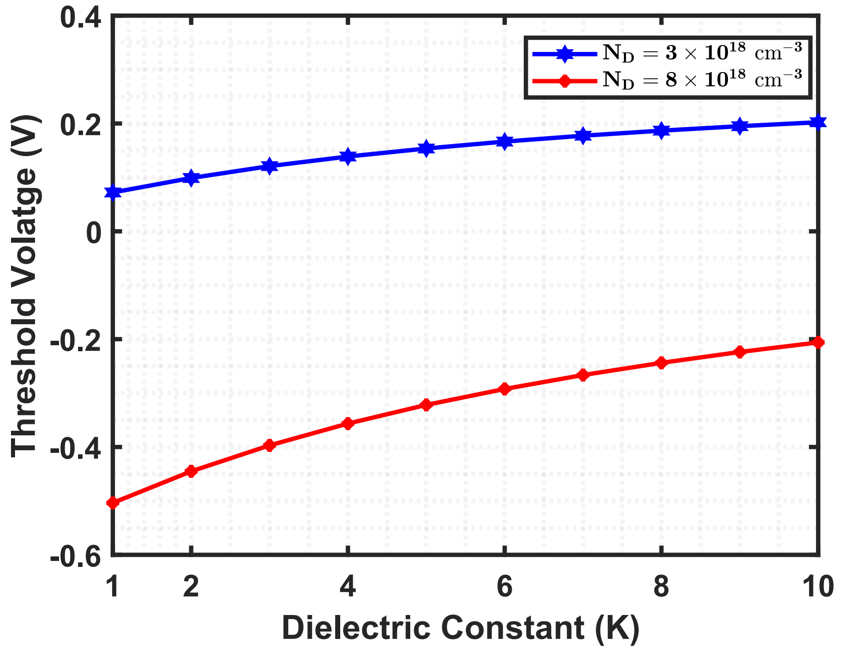

Figure S10: Effect of charge-neutral biomolecules on the threshold voltage with varying dielectric constant within the cavity region for structure E, considering two different doping concentrations, N_D = 3 × 10^{18} cm^{-3} and 8 × 10^{18} cm^{-3}. To ensure comparability, the devices have identical parameters, including a drain-to-source voltage of 1 V, a total gate length of 60 nm, a total cavity length of 30 nm, and the work function of a gate electrode, φ = 4.88 eV.

However, the threshold voltage decreases significantly with increased doping concentration, regardless of the dielectric constant value in the cavity region. This is because the increased doping concentration enhances the channel conductivity by providing more free electrons, thereby reducing the potential barrier for transmitting electrons. Consequently, a lower gate voltage is required to achieve the same current density level when the doping concentration is higher.

Figure S11 shows the change in threshold voltage in structure E for two different doping concentrations, N_D = 3 × 10^{18} cm^{-3} and 8 × 10^{18} cm^{-3}. The analysis reveals an increasing trend in the change in threshold voltage with increasing dielectric constant, aligning with the previously discussed observation.

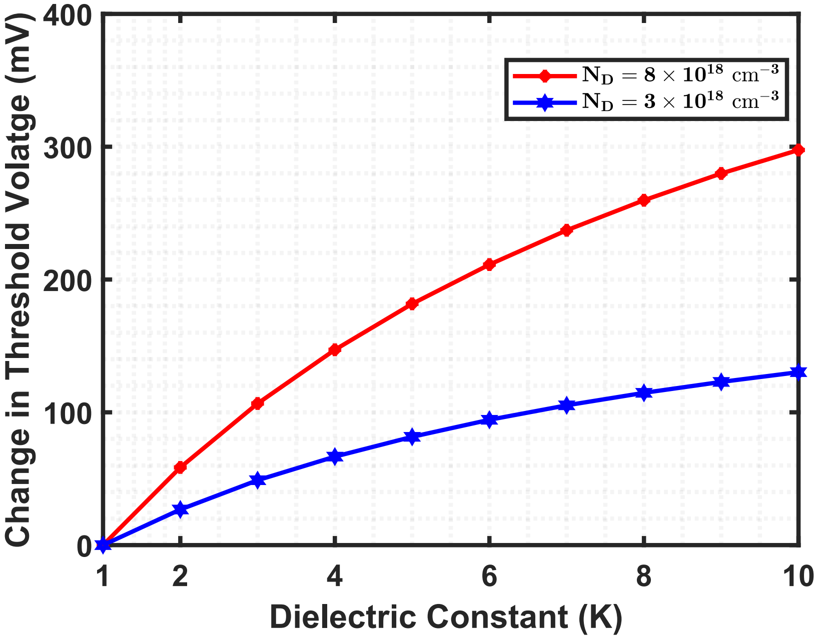

Figure S11: Effect of charge-neutral biomolecules on the change in threshold voltage with varying dielectric constant within the cavity region for structure E, considering two different doping concentrations, $N_D = 3 \times 10^{18}$ cm^{-3} and 8×10^{18} cm^{-3}. To ensure comparability, the devices have identical parameters, including a drain-to-source voltage of 1 V, a total gate length of 60 nm, a total cavity length of 30 nm, and the work function of a gate electrode, φ = 4.88 eV.

Remarkably, it is observed from the figure that, across a wide range of K values, the change in threshold voltage is consistently higher at a higher doping concentration compared to a lower one. For example, at a dielectric constant K = 5, the change in threshold voltage is 181.68 mV for a doping concentration of $N_D = 8 \times 10^{18}$ cm^{-3}, while it is 79.95 mV for a concentration of $N_D = 3 \times 10^{18}$ cm^{-3}.

Figure S12 shows the percentage change in threshold voltage for two different doping concentrations, $N_D = 3 \times 10^{18}$ cm^{-3} and 8×10^{18} cm^{-3}. The observed trend in the percentage change in threshold voltage with respect to the dielectric constant can be explained using reasoning similar to that previously discussed.

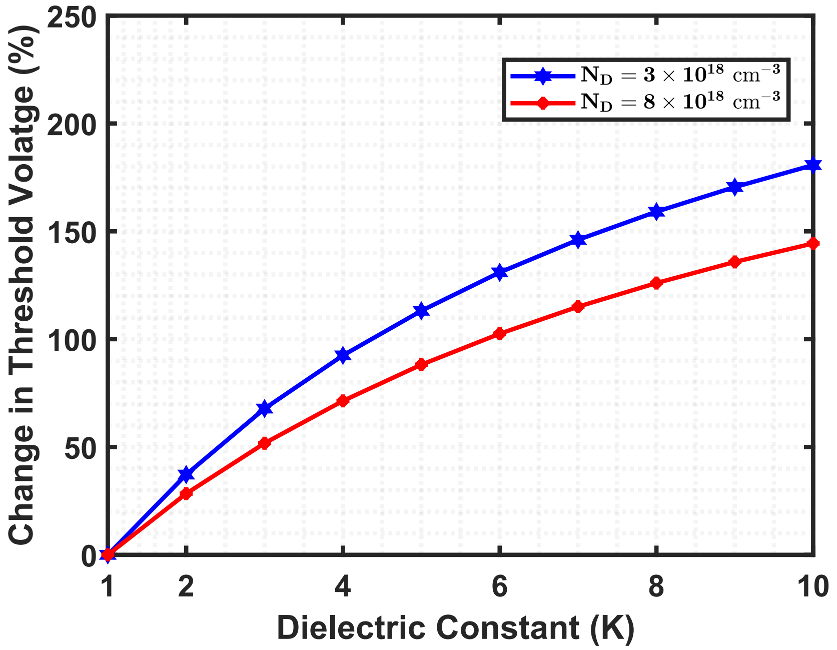

Figure S12: Effect of charge-neutral biomolecules on the percentage change in threshold voltage with varying dielectric constant within the cavity region for structure E, considering two different doping concentrations, $N_D = 3 \times 10^{18}$ cm^{-3} and 8×10^{18} cm^{-3}. To ensure comparability, the devices have identical parameters, including a drain-to-source voltage of 1 V, a total gate length of 60 nm, a total cavity length of 30 nm, and the work function of a gate electrode, φ = 4.88 eV.

Unlike figure S11, which shows a more pronounced change in threshold voltage at higher doping concentrations, this figure presents the opposite trend. The percentage change in threshold voltage has been calculated using equation 2.3. It is notable that, for a dielectric constant K = 1, the absolute value of threshold voltage is greater for $N_D = 8 \times 10^{18}$ cm^{-3}. Consequently, this results in a comparatively smaller percentage change in threshold voltage, while a more significant percentage change is observed for $N_D = 3 \times 10^{18}$ cm^{-3}.

The Ion-off ratio for structure E as a function of the dielectric constant for two different doping concentration values, $N_D = 3 \times 10^{18}$ cm^{-3} and 8×10^{18} cm^{-3}, is shown in figure S13. As discussed earlier, the Ion-off ratio is strongly correlated with the dielectric constant of the cavity region. However, a significant decrease in the Ion-off ratio is observed at higher doping concentrations. This decrease is due to the increased availability of free carriers, which results in a higher off-state current and, consequently, a lower Ion-off ratio.

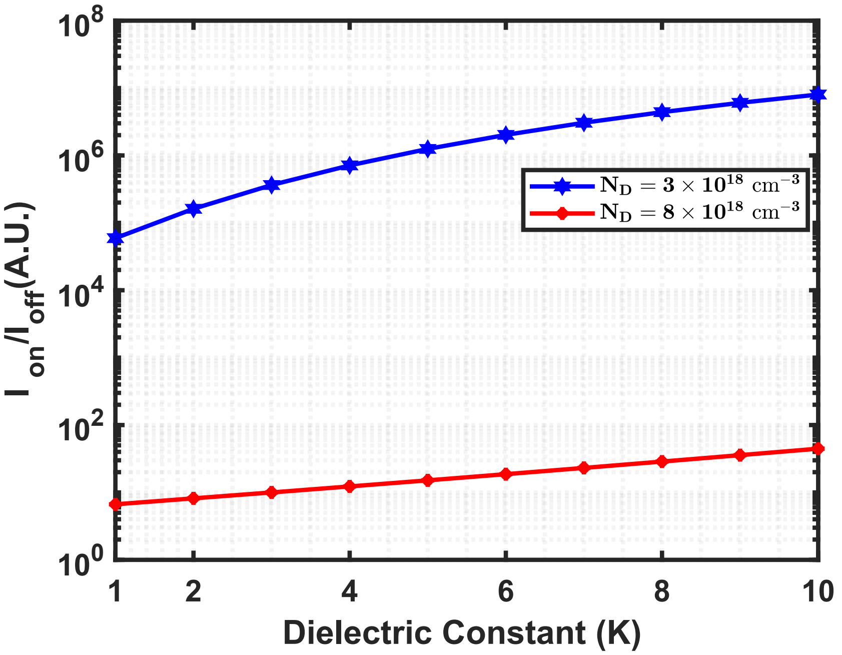

Figure S13: Effect of charge-neutral biomolecules on Ion-off ratio with varying dielectric constant within the cavity region for structure E, considering two different doping concentrations, $N_D = 3 \times 10^{18}$ cm^{-3} and 8×10^{18} cm^{-3}. To ensure comparability, the devices have identical parameters, including a drain-to-source voltage of 1 V, a total gate length of 60 nm, a total cavity length of 30 nm, and the work function of a gate electrode, φ = 4.88 eV.

Figure S14 shows sensitivity as a function of the dielectric constant for two different doping concentration values, specifically $N_D = 3 \times 10^{18}$ cm^{-3} and 8×10^{18} cm^{-3}. The general trend of sensitivity with the dielectric constant in the cavity region can be explained similarly to the previous reasoning. High doping concentrations result in higher channel conductivity due to more free electrons, leading to an increased off-state current. This increase in conductivity reduces the variation of off-state current caused by the presence of biomolecules in the cavity region. Moreover, a higher off-state current is observed at K = 1. As a result, sensitivity is reduced for a high doping concentration across a wide range of K values.

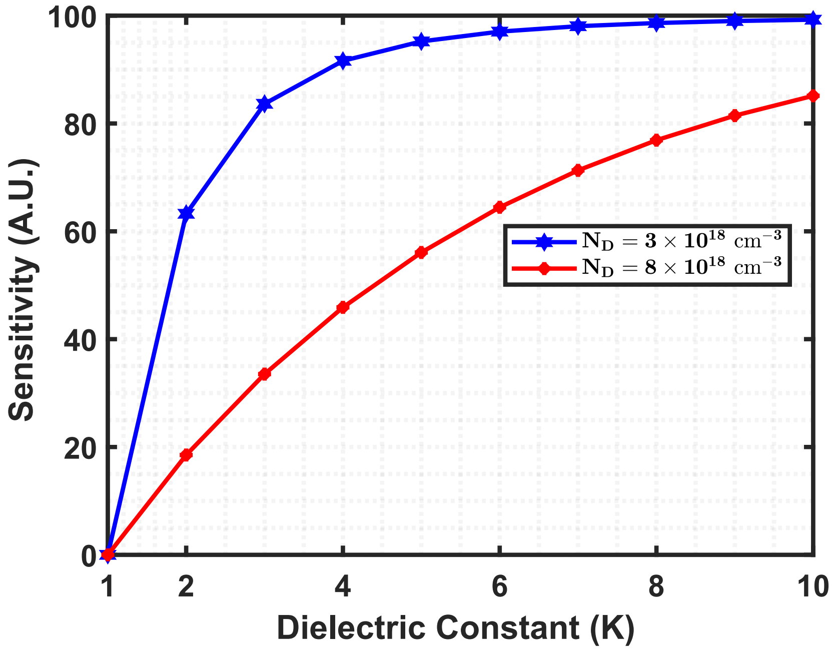

Figure S14: Effect of charge-neutral biomolecules on sensitivity with varying dielectric constant within the cavity region for structure E, considering two different doping concentrations, $N_D = 3 \times 10^{18}$ cm^{-3} and 8×10^{18} cm^{-3}. To ensure comparability, the devices have identical parameters, including a drain-to-source voltage of 1 V, a total gate length of 60 nm, a total cavity length of 30 nm, and the work function of a gate electrode, $\varphi = 4.88$ eV.

The Impact of Gate Length on Biosensing Metrics

Figure S15 presents the drain current variation as a function of the gate voltage for two different gate lengths, $L_G = 60$ and 100 nm. The observed trend is similar to that explained earlier. However, increasing the gate length enhances electrostatic control over the channel potential, resulting in a steeper subthreshold slope and more effective carrier depletion, which reduces the off-state current [4]. Additionally, longer gate lengths mitigate drain-induced barrier lowering (DIBL), leading to a more stable and higher potential barrier near the source, which further decreases the off-state current [5].

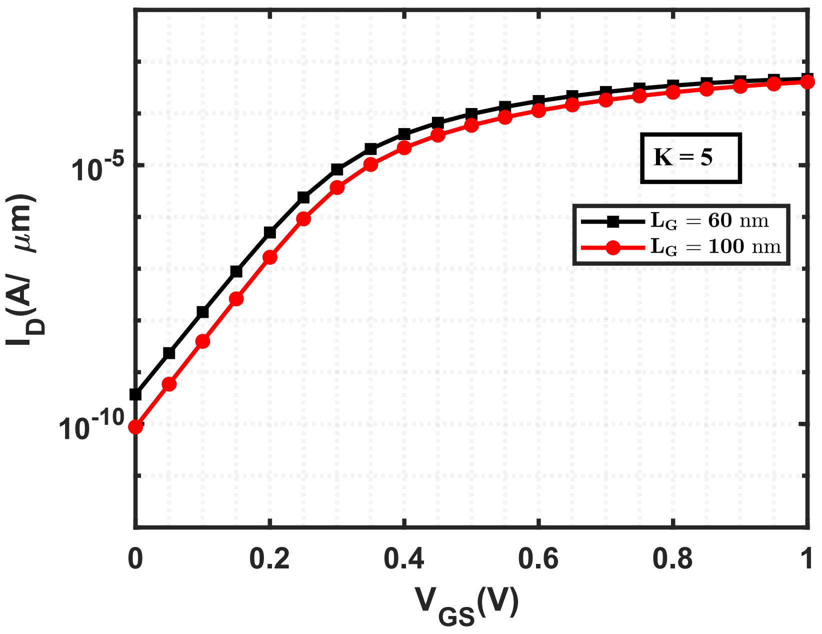

Figure S15: Drain current vs. gate voltage characteristics for two different gate lengths, L_G = 60 and 100 nm. The graphs are shown for structure E with a dielectric constant value of 5. To ensure comparability, the devices have identical parameters, including a drain-to-source voltage of 1 V, a total gate length of 60 nm, the work function of a gate electrode, φ = 4.88 eV, a cavity region comprising 50 % of the gate length, and a doping concentration of $N_D = 3 \times 10^{18}$ cm^{-3}.

Figure S16 shows the threshold voltage as a function of the dielectric constant for two different gate length values, L_G = 60 and 100 nm. The variations in threshold voltage with respect to the dielectric constant, obtained through the constant current method for the cavity region, follow similar principles discussed in the earlier section. For the shorter gate length (L_G = 60 nm), the diminished electrostatic control of the gate over the channel, results in earlier device activation at lower gate voltages. Additionally, DIBL further reduces the threshold voltage [6].

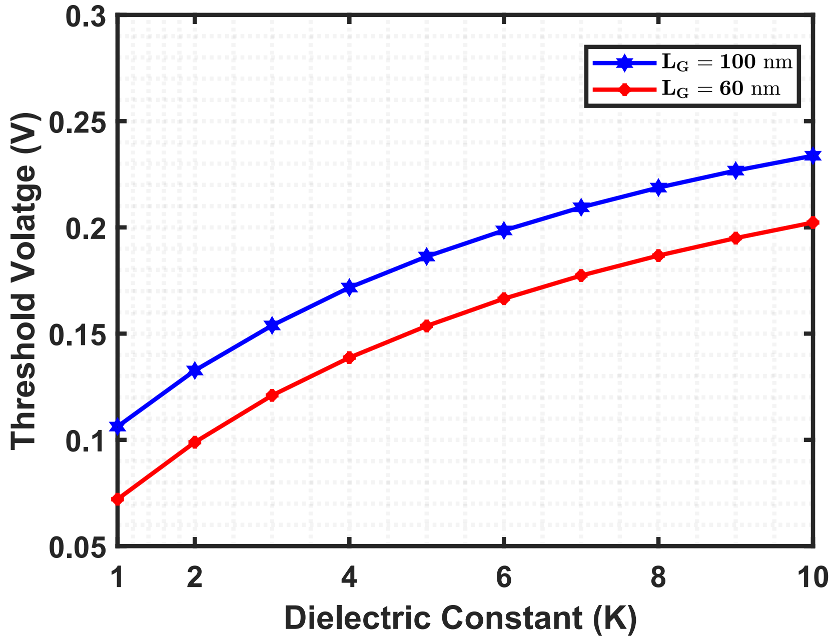

Figure S16: Effect of charge-neutral biomolecules on threshold voltage with varying dielectric constant within the cavity region for structure E, considering two different gate lengths, L_G = 60 and 100 nm. To ensure comparability, the devices have identical parameters, including a drain-to-source voltage of 1 V, a total gate length of 60 nm, the work function of a gate electrode, φ = 4.88 eV, a cavity region comprising 50% of the gate length, and a doping concentration of $N_D = 3 \times 10^{18}$ cm^{-3}.

In contrast, for the longer gate length (L_G = 100 nm), improved gate control over the channel requires a higher gate voltage to reach the threshold condition, as short-channel effects are significantly mitigated. Consequently, a higher threshold voltage is observed for the longer gate length compared to the shorter one.

The change in threshold voltage for two different gate lengths, L_G = 60 and 100 nm, is shown in figure S17. As we observed an increasing trend in threshold voltage for both gate lengths with different K values, the change in threshold voltage followed a trend similar to that previously

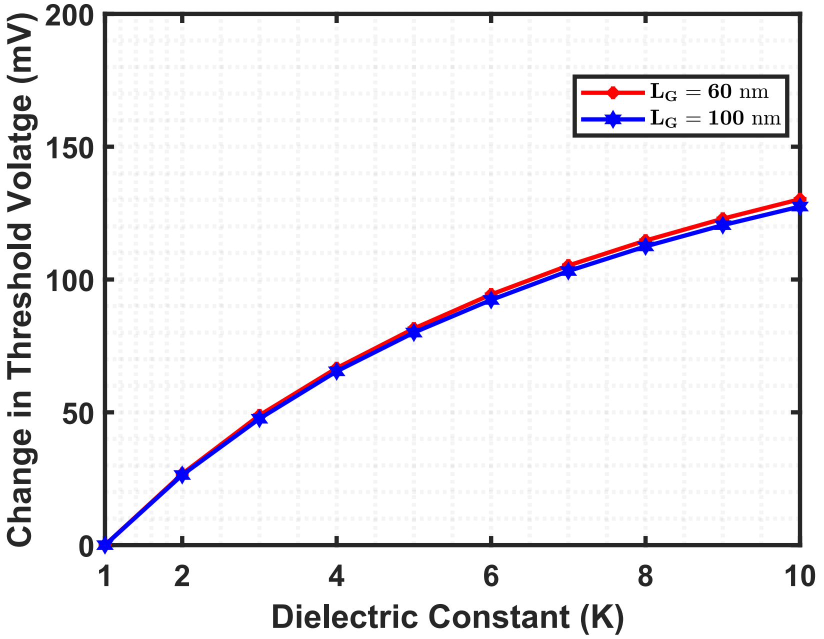

Figure S17: Effect of charge-neutral biomolecules on the change in threshold voltage with varying dielectric constant within the cavity region for structure E, considering two different gate lengths, L_G = 60 and 100 nm. To ensure comparability, the devices have identical parameters, including a drain-to-source voltage of 1 V, a total gate length of 60 nm, the work function of a gate electrode, φ = 4.88 eV, a cavity region comprising 50 % of the gate length, and a doping concentration of $N_D = 3 \times 10^{18}$ cm^{-3}.

discussed. However, it is observed that the change in the threshold voltage remains almost the same up to K = 4 for both gate lengths. Beyond this value, a slightly larger change in the threshold voltage is observed for the shorter gate length compared to the longer one.

Figure S18 shows the percentage change in threshold voltage for two different gate length values, L_G = 60 and 100 nm. The trend of the percent change in the threshold voltage can be explained using the same reasoning as described previously. However, this figure clearly indicates that the percentage change in threshold voltage is more pronounced for a shorter gate length compared to a longer one. For example, at a dielectric constant of K = 10, the percentage change in threshold voltage is 180.72 % for a gate length of L_G = 60 nm, whereas it is 119.96 % for L_G = 100 nm.

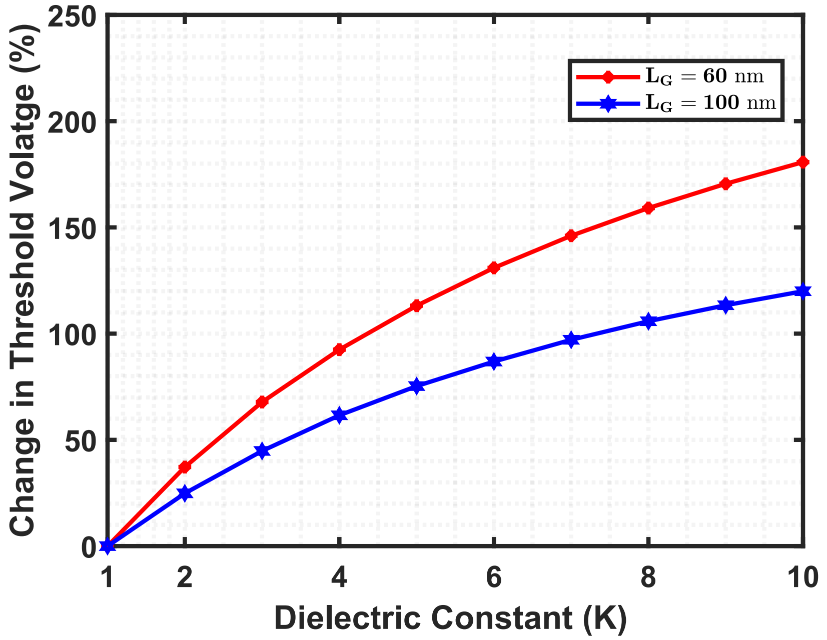

Figure S18: Effect of charge-neutral biomolecules on the percentage change in threshold voltage with varying dielectric constant within the cavity region for structure E, considering two different gate lengths, L_G = 60 and 100 nm. To ensure comparability, the devices have identical parameters, including a drain-to-source voltage of 1 V, a total gate length of 60 nm, the work function of a gate electrode, φ= 4.88 eV, a cavity region comprising 50 % of the gate length, and a doping concentration of $N_D = 3 \times 10^{18}$ cm^{-3}.

Figure S19 shows the Ion-off ratio as a function of the dielectric constant for two different gate length values, L_G = 60 and 100 nm. The correlation between the Ion-off ratio and the dielectric constant of the cavity region has the same explanation as previously stated. As the gate length increases, the off-state current reduction becomes much higher than the on-state current, resulting in a greater Ion-off ratio. Although figure S15 specifically showed the impact of a single dielectric constant, this trend applies to all other dielectric constant values in the cavity region.

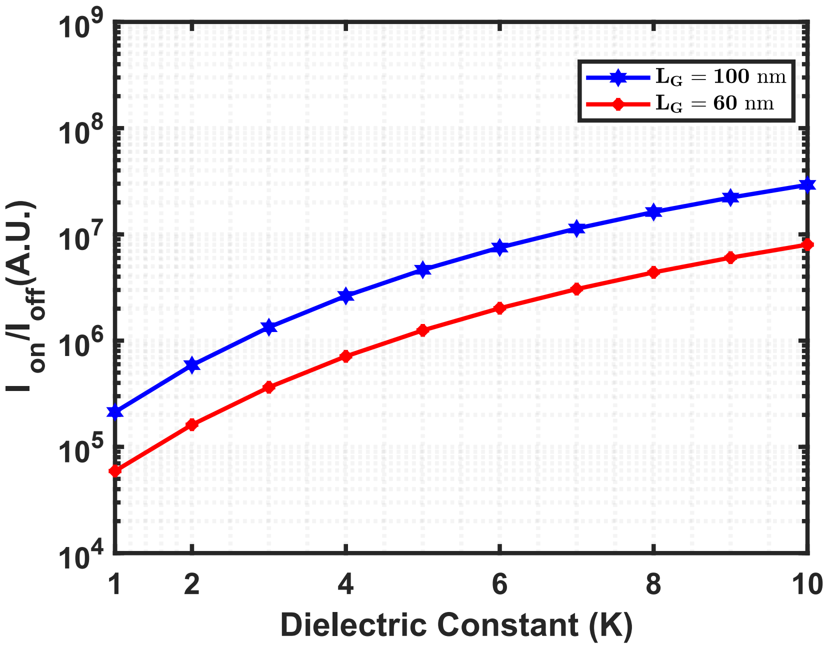

Figure S19: Effect of charge-neutral biomolecules on Ion-off ratio with varying dielectric constant within the cavity region for structure E, considering two different gate lengths, L_G = 60 and 100 nm. To ensure comparability, the devices have identical parameters, including a drain-to-source voltage of 1 V, a total gate length of 60 nm, the work function of a gate electrode, φ = 4.88 eV, a cavity region comprising 50 % of the gate length, and a doping concentration of $N_D = 3 \times 10^{18}$ cm^{-3}.

Figure S20 shows sensitivity as a function of the dielectric constant for two different gate length values, L_G = 60 and 100 nm. The overall correlation between the sensitivity and the dielectric constant has already been explained. It is evident that the gate length has no impact on the sensitivity over a wide range of dielectric constant values. This allows us to design the gate length to improve the performance of other biosensing metrics, as shown before, without compromising sensitivity.

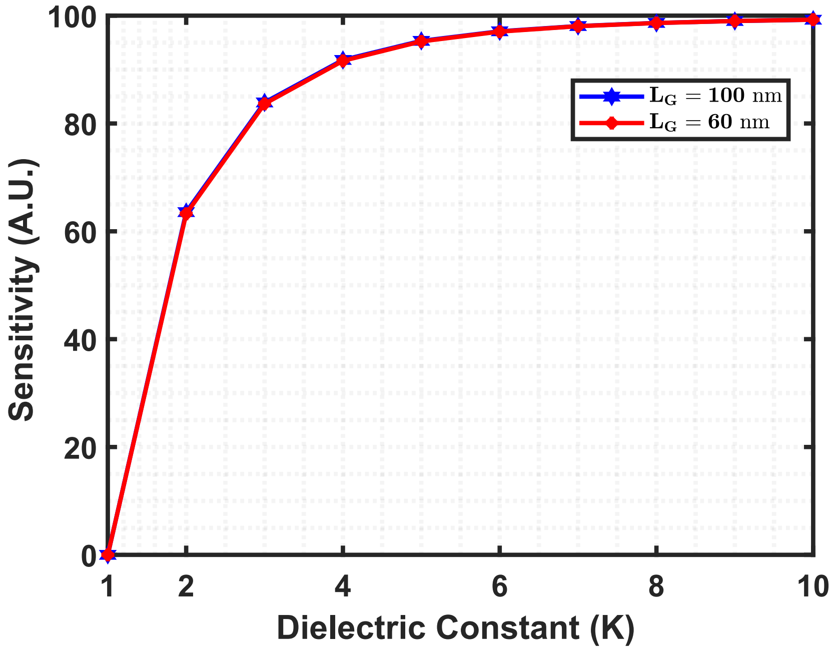

Figure S20: Effect of charge-neutral biomolecules on sensitivity with varying dielectric constant within the cavity region for structure E, considering two different gate lengths, L_G = 60 and 100 nm. To ensure comparability, the devices have identical parameters, including a drain-to-source voltage of 1 V, a total gate length of 60 nm, the work function of a gate electrode, φ = 4.88 eV, cavity region comprising 50 % of the gate length, and a doping concentration of $N_D = 3 \times 10^{18}$ cm^{-3}.

References

1. Neamen DA (2012) Semiconductor Physics and Devices, Fourth Edition. Alphred Rod

2. Angara M, Jena B, Sudandhira Veeran R (2021) Effect of Gate Metal Work-function on Junctionless (JL) Nanowire GAA MOSFET Performance

3. Lim BS, Arshad MKM, Othman N, et al (2014) The impact of channel doping in junctionless field effect transistor. In: 2014 IEEE International Conference on Semiconductor Electronics (ICSE2014). pp 112–114

4. Khanna VK (2016) Short-Channel Effects in MOSFETs. pp 73–93

5. Sahay S, Kumar MJ (2019) Fundamentals of Junctionless Field‐ Effect Transistors. In: Junctionless Field-Effect Transistors: Design, Modeling, and Simulation. pp 67–123

6. Wu M, Jin X, Chuai R, et al (2014) Simulation study on short channel double-gate junctionless field-effect transistors. Journal of Semiconductors 34:34004. https://doi.org/https://doi.org/10.1088/1674-4926/34/3/034004
